# What Shapes RNA Interference Responsiveness in Heteroptera (Insecta: Hemiptera)? An Evidence-Constrained Multilevel Framework for Experimental Design and Optimisation

**DOI:** 10.3390/insects17090900

**Published:** 2026-08-27

**Authors:** Anna Zielińska, Julia Rojek, Jerzy A. Lis

**Affiliations:** 1 Institute of Biology, University of Opole, Oleska 22, 45-052 Opole, Poland; anna.zielinska2@uni.opole.pl; 2 Faculty of Natural Sciences and Technology, University of Opole, Kominka 6, 45-040 Opole, Poland; 134878@student.uni.opole.pl

**Keywords:** double-stranded RNA, Hemiptera, oral RNAi, dsRNA degradation, extracellular nuclease, systemic RNAi, functional genomics, insect pest management

## Abstract

RNA interference (RNAi) is widely used in insect functional genomics and has potential for species-selective pest management, but its performance in Heteroptera varies among species, delivery routes, targets, and endpoints. This review evaluates processes that may constrain double-stranded RNA (dsRNA) delivery, processing, and phenotypic expression using a claim-level evidence hierarchy. Productive RNAi has been demonstrated in heteropteran lineages, particularly after injection, whereas oral and topical outcomes are more variable. Responses after oral, plant-mediated, and carrier-assisted delivery have also been reported in Miridae, although evidence remains concentrated in Pentatomidae. Extracellular degradation is the best-documented candidate constraint, but causal evidence linking a specific nuclease to oral RNAi remains restricted to *Nezara viridula*, and the in vivo relevance of haemolymph degradation is unresolved. Distant-tissue and systemic effects provide functional evidence of signal access, whereas restrictions imposed by the perimicrovillar membrane, limited cellular uptake, endosomal entrapment, and inefficiency at Dicer or RISC steps remain unresolved as limiting mechanisms. We propose an evidence-constrained multilevel framework that treats the delivery route as an experimental perturbation and transcript depletion, protein reduction, and phenotype as distinct outcomes. Mechanism-matched measurements and staged causal tests are required to identify context-specific constraints and guide optimisation.

## Simple Summary

Many true bugs (Heteroptera), including several major crop pests, are difficult to control. RNA interference (RNAi) can reduce the activity of a selected gene and is useful for studying insect biology and developing selective pest-control methods. In many experiments, RNAi is effective when double-stranded RNA is injected but weaker or less predictable after feeding or application to the insect surface. We reviewed evidence for the biological processes that may explain this variation. The strongest direct evidence concerns the breakdown of double-stranded RNA outside cells, particularly in saliva and digestive fluids of selected true bugs, including Pentatomidae and Miridae. Functional silencing in tissues distant from the exposure site has also been observed, although the nature and movement of the silencing signal remain unresolved. Other proposed explanations, including restricted passage across the gut, poor cellular entry, trapping within cellular compartments, and inefficient intracellular processing, have not been demonstrated as limiting steps in true bugs. We therefore present an evidence-constrained framework for identifying processes that limit RNAi in a defined species, life stage, delivery route, tissue, and target-gene system. Comparing delivery routes and measuring intermediate steps should improve experimental interpretation and guide more reliable, species-specific pest-management approaches.

## 1. Introduction

RNA interference (RNAi) is a conserved mechanism of sequence-specific gene regulation mediated by small RNAs. In the exogenous small interfering RNA (siRNA) pathway, double-stranded RNA (dsRNA) is processed into siRNAs that guide the RNA-induced silencing complex (RISC) to complementary messenger RNAs, resulting in cleavage of the target transcript. Since RNAi was first described in *Caenorhabditis elegans*, it has become an established tool for functional genomics and a potential platform for species-selective pest management [1,2].

The applied potential of RNAi is particularly well illustrated in several coleopteran pests, where orally delivered dsRNA can induce substantial target-transcript depletion and mortality [2,3,4]. These findings have supported the development of host-induced gene silencing (HIGS), spray-induced gene silencing (SIGS), and carrier-assisted delivery systems. They have also demonstrated that RNAi performance cannot be predicted from sequence complementarity or taxonomic affiliation alone. Extracellular persistence, exposure to responsive tissues, cellular internalisation, intracellular processing, target-gene biology, and the selected molecular or phenotypic endpoint can each influence the observed outcome [2,4,5,6].

In the United States, this potential has progressed to commercial deployment against coleopteran pests. SmartStax PRO maize expresses a dsRNA construct targeting *DvSNF7* in *Diabrotica virgifera virgifera* J. L. LeConte, 1868, whereas Calantha is a foliar-applied product containing the dsRNA active ingredient ledprona, developed against *Leptinotarsa decemlineata* (Say, 1824) [7]. These products demonstrate that both plant-incorporated and exogenously applied dsRNA can be translated into crop-protection technologies when target-species susceptibility, route of exposure, and dsRNA persistence are sufficiently favourable. They do not, however, imply that comparable performance should be expected in Heteroptera [7].

This variability is especially relevant to Hemiptera, an economically important order containing numerous crop pests and vectors of plant pathogens. Within Heteroptera (Insecta: Hemiptera), target-transcript depletion and target-linked phenotypes have been demonstrated in model species and pests from several families, but the magnitude and reproducibility of these responses vary by target, developmental stage, delivery route, and experimental design. The suborder also encompasses substantial diversity in feeding mode, gut organisation, development, and target-tissue physiology, making broad inference from a small number of intensively studied pentatomids particularly uncertain. Injection frequently produces stronger or more consistent responses than oral or topical delivery in directly compared experimental systems, although this pattern is neither universal nor sufficient to identify the responsible process [5,6,8]. Injection-induced target-transcript depletion demonstrates that the construct can engage the silencing pathway after some route-specific processes have been bypassed; it does not establish optimal intracellular efficiency, uniform tissue distribution, or the mechanism limiting another delivery route.

The evidence supporting individual candidate constraints remains markedly uneven. Extracellular dsRNA degradation has been measured directly in saliva, gut fluid, or haemolymph in selected members of Pentatomidae and Miridae [9,10,11,12,13,14,15,16,17]. In *Lygus lineolaris* (Palisot de Beauvois, 1818), dsRNA degradation was detected in saliva, whereas degradation in haemolymph occurred more slowly under the applied ex vivo assay conditions [16]. In *Apolygus lucorum* (Meyer-Dür, 1843), dsRNA degradation was observed in midgut fluid, and purified AldsRNase exhibited nuclease activity against dsRNA [17]. These findings broaden the taxonomic scope of direct evidence of extracellular degradation but do not establish a causal relationship between the identified activities and RNAi performance. Causal evidence linking perturbation of a specific nuclease to an improved oral RNAi outcome remains restricted to *Nezara viridula* (Linnaeus, 1758) [12].

Functional RNAi responses have nevertheless been obtained in Miridae under several exposure regimes, including plant-mediated delivery, chitosan-assisted oral delivery, and SPc-assisted topical or mixed-route exposure [18,19,20]. These outcomes support the functional completion of the delivery sequence in the respective experimental systems but do not directly resolve cellular internalisation or the contribution of individual delivery steps. Functional responses in distant tissues and systemic or maternal effects have further shown that silencing can extend beyond the site of administration in particular species and experimental contexts [21,22,23,24]. Such observations provide functional evidence that distant or developing tissues can be affected but do not identify the molecular form or route of the biologically active signal, nor quantify its abundance.

By contrast, several frequently proposed mechanisms remain insufficiently tested in Heteroptera. The perimicrovillar membrane (PMM) is anatomically documented in multiple heteropteran taxa, but its role in restricting dsRNA passage has not been examined directly [25,26,27,28,29]. Core components of the siRNA pathway have been identified in heteropterans, and processing of dsRNA into siRNA-sized products has been observed in selected species [9,15,30]. Nevertheless, deficiencies in individual Dicer-2- or RISC-associated processes have not been shown to be limiting mechanisms. Insufficient cellular internalisation and endosomal retention have likewise not been shown to constrain heteropteran RNAi outcomes. These processes should therefore be treated as biologically plausible diagnostic hypotheses, informed partly by evidence from other insects, rather than as established barriers across the suborder [2,5,6]. A multilevel model is analytically useful only when these differences in evidential status remain explicit.

Interpretation also requires separating successive molecular and biological outcomes. Target-transcript depletion, when established using appropriate sequence and procedural controls, provides evidence of sequence-specific silencing but does not demonstrate proportional depletion of the encoded protein or the emergence of an organismal phenotype. Conversely, mortality or another phenotype without corresponding molecular confirmation cannot by itself establish target-specific RNAi. The absence of an expected phenotype therefore cannot, on its own, be taken as evidence of an unsuitable target, a non-responsive species, or failure of the entire RNAi pathway. Delivery route, dose, tissue exposure, sampling time, protein turnover, functional redundancy, and the threshold required to produce the phenotype must also be considered [2,5,6].

Previous reviews have addressed insect RNAi mechanisms, applications in pest management, and the challenges associated with RNAi in hemipteran insects [2,5,6,8]. This review focuses specifically on Heteroptera and evaluates the biological processes and candidate constraints that may shape RNAi responsiveness from extracellular exposure through intracellular processing to molecular and phenotypic outcomes. It applies an evidence-constrained multilevel framework that distinguishes direct causal evidence, direct process observations, functional evidence, candidate-level inference, comparative mechanisms, and unresolved steps. The objective is not to assign a universal barrier profile to Heteroptera, but to support more defensible interpretation, experiments matched to the supported constraint, and route-specific optimisation in functional genomics and future pest-management research.

### Review Scope and Literature Selection

This review was designed as a narrative, conceptually oriented synthesis rather than a systematic review or meta-analysis. Its purpose was to integrate evidence relevant to the variable performance of experimentally induced RNAi in Heteroptera and to evaluate the degree of support for individual mechanistic claims. No formal risk-of-bias assessment, effect-size estimation, or quantitative synthesis was undertaken. Evidence was assessed at the level of a specific claim concerning a biological process or experimental outcome rather than at the level of the publication as a whole; the same study could therefore support different claims at different evidence levels.

Literature searches were conducted primarily in the Web of Science Core Collection, Scopus, and PubMed, supplemented by targeted searches in Google Scholar and screening of reference lists. The search was last updated in August 2026. Search terms included combinations of RNA interference, RNAi, double-stranded RNA, dsRNA, siRNA, Hemiptera, Heteroptera, delivery route, extracellular nuclease, dsRNA stability, systemic RNAi, cellular uptake, endocytosis, endosomal escape, and core components of the siRNA pathway. Additional searches combined these terms with relevant heteropteran family and species names. Targeted species searches included *Halyomorpha halys*, *Nezara viridula*, *Euschistus heros*, *Piezodorus guildinii*, *Plautia stali*, *Cimex lectularius*, *Rhodnius prolixus*, *Oncopeltus fasciatus*, *Apolygus lucorum*, and *Lygus lineolaris*.

Priority was given to peer-reviewed primary studies reporting functional RNAi, dsRNA stability, tissue-resolved outcomes, or genomic and transcriptomic characterisation relevant to the exogenous siRNA pathway in Heteroptera. Evidence from other hemipterans and other insect orders was used only when it informed a testable comparative mechanism for a process not directly resolved in Heteroptera. Such evidence was kept separate from heteropteran evidence and did not increase the evidence class assigned to the process in Heteroptera.

Evidence was classified at the claim level as follows: A1, direct mechanistic evidence with a causal perturbation; A2, direct mechanistic observation without a decisive perturbation; B, direct functional evidence showing that the pathway operates without identifying the responsible step; C, indirect or candidate-level evidence; D, a mechanism demonstrated in another insect order and used only to formulate a heteropteran hypothesis; and E, an unresolved process lacking a direct test in Heteroptera. Table 1 presents selected representative functional studies and is intended to illustrate taxonomic, delivery, target, and endpoint diversity rather than constitute an exhaustive quantitative dataset. Table 2 applies the evidence hierarchy to specific processes considered in the review.

## 2. RNAi Responsiveness in Heteroptera: Functional Evidence and Context Dependence

Experimentally induced RNAi responses have been reported in species representing several heteropteran families, and RNAi has been used as a functional genomics approach in both model and pest species [2,6,9,10,11,12,13,14,16,18,19,20,21,22,23,30,32,33,34,35,36,37]. In the species subjected to genomic or transcriptomic analysis, candidate homologues of Dicer and Argonaute, as well as proteins potentially involved in dsRNA binding or handling, have been identified [9,30]. These annotations indicate a molecular repertoire compatible with the exogenous siRNA pathway but do not establish its expression or activity in every relevant tissue, the catalytic efficiency of individual components, effective small-RNA loading, or uniform pathway activity. Functional competence is supported more directly by depletion of the intended target transcript under appropriate experimental controls. Concordant changes in the encoded protein, protein activity, or a biologically linked phenotype can strengthen causal interpretation but do not replace molecular confirmation of target-transcript depletion.

A substantial proportion of the functional evidence derives from injection-based experiments. Embryonic or post-embryonic injection in *Oncopeltus fasciatus* (Dallas, 1852) produced reproducible gene-associated developmental phenotypes, although target-transcript depletion was not measured. Injection in *Rhodnius prolixus* Stål, 1859, *Cimex lectularius* Linnaeus, 1758, and several pentatomids has produced target-transcript depletion, sometimes accompanied by reproductive, physiological, developmental, or survival effects [9,10,13,21,23,30,31,32,33,34,35,36,37]. These findings demonstrate functional engagement of RNAi in the specific species–stage–construct–target combinations examined. They do not establish equivalent responsiveness across tissues or species, optimal efficiency of every intracellular pathway step, or delivery of sufficient intact dsRNA to all relevant target tissues. Post-embryonic haemocoelic injection bypasses salivary, gut-lumen, and midgut-interface processes but retains potential losses associated with haemolymph persistence, tissue distribution, cellular internalisation, and intracellular processing.

The evidence base remains taxonomically and experimentally uneven. Representative studies summarised in Table 1 include members of Pentatomidae, Cimicidae, Reduviidae, Lygaeidae, and Miridae. Within Miridae, functional responses have been reported following plant-mediated exposure in *A. lucorum*, chitosan-assisted feeding, and SPc-assisted direct topical or mixed-route exposure [18,19,20]. Nevertheless, pentatomids and a small number of established model species account for a disproportionate share of the available evidence. Delivery methods, administered and biologically available doses, developmental stages, target genes, sampling schedules, molecular assays, and definitions of a positive outcome differ substantially among studies. The current literature therefore shows that experimentally induced RNAi can operate across multiple heteropteran lineages but does not provide a suborder-wide estimate of susceptibility or a defensible ranking of species according to RNAi responsiveness.

In several within-study comparisons, injection produced greater or more reproducible target-transcript depletion or phenotypic effects than oral or topical exposure [10,13,32,33]. This pattern is experimentally informative but mechanistically non-specific. A positive injection response indicates that the construct and target can support silencing when oral or cuticular interfaces are bypassed. It does not determine whether a weaker response through another route results from extracellular degradation, limited epithelial passage, altered tissue distribution, insufficient cellular internalisation, intracellular losses, or inadequate biological exposure. Cross-study comparisons remain less decisive because nominal doses administered via different routes rarely correspond to equivalent amounts of intact dsRNA reaching the relevant tissues.

The findings for *L. lineolaris* provide an additional example of route-dependent performance. The same *IAP* target and long-dsRNA construct had produced an RNAi response following injection in an earlier study, whereas oral delivery of ds*IAP* or *IAP*-derived siRNA in membrane sachets produced no apparent transcript reduction by semi-quantitative RT-PCR and no target-specific survival effect, despite confirmed food uptake [16]. However, the injection and feeding experiments were conducted separately and were not dose-matched. The difference between their outcomes therefore cannot be attributed exclusively to salivary degradation or to any other route-associated process.

Oral RNAi outcomes range from measurable depletion of the target transcript, with or without an associated phenotype, to weak or undetectable molecular responses. Functional oral responses have been reported in several pentatomids following delivery of naked or formulated dsRNA [10,11,13,14,32]. In *A. lucorum*, feeding on transgenic maize and soybean expressing dsRNA against *V-ATPase-E* resulted in target transcript depletion and increased mortality, providing evidence of plant-mediated RNAi [19]. Feeding on bean fragments treated with chitosan-complexed dsRNA targeting *GRK2* likewise produced target transcript depletion accompanied by increased mortality, reduced body mass, and prolonged development [20]. These studies demonstrate that oral or plant-mediated exposure can complete the delivery sequence sufficiently to generate molecular and phenotypic responses in particular experimental systems. They do not establish equivalent internal exposure across treatments or identify the individual extracellular, epithelial, cellular, or intracellular processes responsible for differences in outcome. Greater target-transcript depletion need not produce a proportional phenotypic effect, whereas mortality without corresponding molecular confirmation cannot establish target-specific RNAi.

Evidence for topical RNAi remains limited and heterogeneous. In *C. lectularius*, injection produced a functional response, whereas topical application did not [33]. In *Halyomorpha halys* (Stål, 1855), outcomes varied among target–dose–time combinations, and molecular and survival endpoints were not consistently concordant [22]. By contrast, an SPc-complexed dual-target formulation evaluated in *A. lucorum* produced target-transcript depletion and increased nymphal mortality following direct topical application and under a spraying regime in which the formulation was applied to bean plants bearing nymphs [18]. The direct topical treatment demonstrates functional completion of the delivery sequence under the tested species–stage–target conditions. The spraying treatment demonstrates efficacy under a mixed-exposure regime but does not distinguish direct contact from acquisition through treated plant material or other exposure pathways. Collectively, the available studies are insufficient to establish the general feasibility of topical RNAi or to determine whether cuticular properties constitute a common limiting mechanism across Heteroptera.

The heteropteran literature therefore supports a continuum of context-dependent RNAi outcomes rather than a binary division between responsive and refractory species. A study may demonstrate target-transcript depletion without a corresponding phenotype, a phenotype whose magnitude depends on developmental or physiological background, or route-dependent differences without identifying the responsible process. RNAi responsiveness should consequently be defined for a specified species, developmental stage, delivery route, formulation, construct, biological exposure, target tissue, sampling schedule, and molecular or phenotypic endpoint. The following sections examine the extracellular, physiological, cellular, and intracellular processes that may shape this response while maintaining a distinction among directly demonstrated mechanisms, functional inferences, and unresolved candidate constraints.

## 3. Extracellular Degradation and Stability Constraints

Extracellular degradation can reduce the fraction of administered dsRNA that remains intact long enough to reach responsive cells, particularly after oral delivery. In He-teroptera, direct evidence remains concentrated in a small number of species but is not restricted to Pentatomidae. Salivary, gut-fluid, and haemolymph-associated degradation has been documented in selected pentatomids [9,10,11,12,13,14,15]. Within Miridae, *L. lineolaris* exhibited salivary dsRNA degradation and slower degradation in haemolymph under the applied ex vivo assay conditions [16], whereas dsRNA degradation in midgut fluid and dsRNA-degrading activity of purified AldsRNase were demonstrated in *A. lucorum* [17]. These observations provide direct evidence of extracellular degradation or purified-nuclease activity but do not, by themselves, establish the contribution of the detected activity to an RNAi outcome. Causal evidence showing that perturbation of a nuclease can improve oral RNAi remains restricted to *N. viridula* [12]. Interpretation therefore requires distinguishing between detecting degradation in an isolated biological matrix, showing that a specific nuclease contributes to that activity, demonstrating formulation-dependent protection, and establishing that preservation of dsRNA improves a downstream RNAi outcome.

Orally administered dsRNA may encounter salivary and digestive fluids before any potential passage across the gut epithelium, whereas injection bypasses these early exposures and delivers dsRNA directly into the haemocoel, where it may remain susceptible to haemolymph-associated degradation [10,11,12,13,14,16]. Accordingly, a difference between oral and injection-based responses is consistent with a route-associated loss occurring before cytoplasmic processing, but it does not identify the responsible compartment or mechanism. The following subsections therefore evaluate salivary, gut-fluid, and haemolymph-associated degradation separately and restrict each conclusion to the taxonomic and experimental context in which it was obtained.

### 3.1. Salivary Degradation of dsRNA

During feeding by phytophagous heteropterans, dsRNA may mix with salivary secretions before and during ingestion. Direct evidence of salivary dsRNA degradation is presently available for selected pentatomids, including *N. viridula*, *Euschistus heros* (Fabricius, 1798), and *H. halys*, and for the mirid *L. lineolaris* [10,11,12,13,14,16].

In *N. viridula*, untreated saliva from adults degraded dsRNA almost completely within 10 min and completely within 30 min ex vivo, whereas dsRNA remained relatively stable for 48 h in diet recovered after feeding by nymphs [10]. This contrast shows that the observed degradative activity depends on the assay matrix and biological context, but it does not quantify salivary loss during the nymphal bioassay. Stronger causal evidence was obtained for *NvdsRNase*: injection-mediated suppression of this nuclease prolonged dsRNA stability in both saliva and midgut juice and increased mortality following the subsequent oral administration of ds*αCop* from 46.7% to 65% [12]. Because the same intervention affected both matrices, the experiment demonstrates that *NvdsRNase* contributes to oral RNAi performance but does not apportion this contribution between the salivary and digestive compartments.

In *E. heros*, naked dsRNA was completely degraded within 10 min in undiluted saliva. EDTA preserved detectable dsRNA for up to 120 min, whereas liposome encapsulation afforded only partial protection; both formulations increased mortality for some gene targets [11]. However, transcript depletion and mortality were not consistently aligned across targets and formulations. The biological effects therefore cannot be attributed exclusively to salivary protection, nor does the study demonstrate improved cellular uptake or endosomal release.

Results for *H. halys* vary across experimental protocols. Mogilicherla et al. [13] found that saliva did not completely degrade dsRNA within 1 h and exhibited lower degradative activity than that measured in saliva from *Heliothis virescens* (Fabricius, 1777). By contrast, Amineni et al. [14] reported dsRNA degradation within 1 min in both saliva and salivary gland extracts; co-formulation with double-stranded DNA (dsDNA) reduced this degradation and enabled significant *HhCHC* transcript depletion after oral delivery, although mortality was not increased. The studies differed in developmental stage, sample collection, matrix preparation, exposure time, and substrate-to-enzyme ratio, but the contribution of these factors to the discrepant results has not been tested directly. Accordingly, the available evidence does not permit the difference to be assigned to any particular biologi-cal or methodological factor. Moreover, *HhNSE* was proposed based on its expression profile and remains an expression-based candidate nuclease because its causal involvement was not tested through gene-specific suppression or a purified-enzyme assay [14].

In *L. lineolaris*, dsRNA degradation by both directly collected saliva and salivary gland extracts increased with incubation time and biological material concentration [16]. dsRNA was also degraded in the contents of membrane sachets recovered after insect feeding, whereas the tested dsDNA did not undergo analogous degradation. However, the recovered sachet contents were incubated overnight before analysis; consequently, this experiment does not establish the rate of dsRNA degradation during feeding itself. The tested inhibitor did not prevent degradation, but because it targeted single-stranded RNases, its ineffectiveness cannot be interpreted as evidence that the relevant dsRNA-degrading activity is generally resistant to inhibitors. In the associated feeding experiments, neither long ds*IAP* nor *IAP*-derived siRNA produced an apparent reduction in target-transcript abundance by semi-quantitative RT-PCR or a target-specific survival effect, despite confirmed food uptake. Because the nucleolytic activity was not perturbed experimentally, its co-occurrence with the oral non-response does not demonstrate that salivary degradation caused the absence of detectable RNAi.

In *A. lucorum*, *AldsRNase* exhibited high expression in the salivary glands, supporting its designation as a candidate salivary nuclease [17]. However, salivary activity was not examined directly, and secretion of AldsRNase into saliva was not demonstrated. The expression pattern therefore constitutes candidate-level evidence and should not be treated as direct evidence of salivary dsRNA degradation.

Salivary dsRNA degradation has thus been directly demonstrated in several Pentatomidae and one Miridae. Within the evidence hierarchy applied in this review, A1 support remains restricted to *N. viridula*, in which nuclease perturbation improved a downstream oral RNAi outcome [12]. The quantitative in vivo contribution of salivary degradation remains unresolved beyond the specific species, developmental stages, and experimental systems examined. Stage-matched kinetic assays, recovery of intact dsRNA during feeding, and selective perturbation of candidate nucleases are required to relate ex vivo degradation to the biologically available dose reaching the digestive tract.

### 3.2. Gut-Fluid Degradation of dsRNA

Gut-fluid degradation should be distinguished from the broader observation that oral RNAi is often weaker than injection-based RNAi. In *N. viridula*, dsRNA remained largely intact in adult midgut juice after 10 and 30 min, with clear degradation observed after 60 min; under the same experimental conditions, degradation was slower than in untreated adult saliva [10]. Suppression of *NvdsRNase* subsequently prolonged dsRNA stability in midgut juice and increased mortality following oral delivery of ds*αCop* [12]. These results demonstrate nuclease-associated degradation in the tested gut-fluid matrix and provide causal evidence that *NvdsRNase* influences oral RNAi performance. However, because its suppression also reduced salivary degradation, the independent contribution of the gut compartment cannot be quantified from this experiment.

In *A. lucorum*, native midgut fluid directly degraded dsRNA under the applied ex vivo conditions [17]. Purified AldsRNase degraded dsRNA, siRNA, and dsDNA, demonstrating broad nucleolytic activity against the tested substrates. AldsRNase was also localised to the cytoplasm of midgut cells. However, the enzyme was not perturbed experimentally, and its secretion into the gut lumen was not demonstrated. The degradation observed in native midgut fluid therefore cannot be attributed specifically to AldsRNase, nor can the contribution of this enzyme to oral RNAi performance be established. These findings constitute direct mechanistic observations and candidate-level linkage, corresponding to class A2 rather than A1 evidence.

Chitosan-complexed dsRNA used in *A. lucorum* was protected against degradation by RNase A and showed increased stability under the physicochemical conditions examined [20]. However, protection was not tested in native midgut fluid. The study therefore demonstrates resistance to the model nuclease and increased stability under the tested environmental conditions but does not establish protection against the actual mixture of nucleases and other components present in the gut lumen. The subsequent target-transcript depletion and phenotypic effects provide functional evidence of chitosan-assisted oral RNAi, but they do not identify protection from gut-fluid degradation as the cause of the improved response.

Direct evidence of formulation-dependent protection within a native gut matrix was obtained in a subsequent study of *A. lucorum*. Naked dsGFP was rapidly degraded during a 1.5 h incubation with a 50% midgut-fluid preparation, whereas SPc-complexed dsGFP remained detectable under the same conditions [18]. This comparison directly demonstrates degradation of naked dsRNA and SPc-mediated protection in the tested midgut-fluid matrix. However, SPc also affected surface spreading, plant uptake, and route-level dsRNA delivery. The subsequent target-transcript depletion and mortality therefore cannot be attributed exclusively to protection against gut-fluid degradation. Moreover, the spraying bioassay did not distinguish direct contact from ingestion or other exposure through treated plant material.

In *Plautia stali* Scott, 1874, injection induced strong silencing, whereas oral delivery required higher doses and produced weaker responses [32]. Co-feeding dsRNA against the candidate nuclease *PsdsRNase* did not improve silencing of a second target. This negative result does not conclusively rule out nuclease involvement because effective suppression of *PsdsRNase* via the same oral route was not demonstrated. The route comparison therefore indicates that one or more constraints operated upstream of productive intracellular processing but does not identify gut-fluid degradation as the responsible mechanism.

A recent cross-order synthesis shows that dsRNase expression, activity, and effects on RNAi vary across tissues and insect lineages and that genetic suppression or physical protection can improve dsRNA persistence in some systems [38]. These comparisons provide a rationale for diagnostic assays but do not extend the heteropteran evidence base. Gut-fluid degradation has been demonstrated directly in *N. viridula* and *A. lucorum*. However, causal evidence that perturbation of a specific nuclease improves an oral RNAi outcome remains restricted to *N. viridula* [10,12,17,18]. The quantitative importance of gut-fluid degradation in vivo and its generality across Heteroptera remain insufficiently resolved. Addressing these questions requires stage-matched recovery of intact dsRNA, separate analysis of salivary and gut fluids, confirmation of candidate-nuclease secretion, and validated perturbations whose proximal effects can be distinguished from broader formulation-dependent changes.

### 3.3. Haemolymph Nucleases and Systemic Stability

Once dsRNA enters the haemocoel, its extracellular stability can influence the interval available for tissue contact and cellular uptake. Direct ex vivo evidence of haemolymph-associated degradation is available for selected Pentatomidae and Miridae. In *E. heros* haemolymph, dsRNA degradation was partial after 10 min, increased over 30–60 min, and was complete by 120 min [9]. In *H. halys*, dsRNA remained detectable after 1 h in one study [13], whereas another study detected intact dsRNA for up to 2 h but also reported an increasing abundance of degradation products and differences among developmental stages [14]. In *L. lineolaris*, no visible dsRNA degradation was observed after 1 h of incubation with haemolymph, whereas degradation was evident after overnight incubation [16]. Haemolymph-associated degradation was therefore slower than salivary degradation under the conditions of the applied assays. This qualitative difference does not permit a quantitative comparison of nuclease activity based on gel-band intensity. The *L. lineolaris* findings constitute A2 evidence of degradation in an isolated biological matrix, without causal linkage to an RNAi outcome.

Comparative body-fluid assays have also revealed variable nuclease activity in several heteropterans, although the sampled fluid was not always specifically identified as haemolymph [15]. Such findings cannot be uniformly interpreted as direct evidence of haemolymph-associated degradation. More generally, the available ex vivo experiments demonstrate that heteropteran haemolymph can contain dsRNA-degrading activity, but they do not establish the in vivo half-life of dsRNA. Comparisons between degradation in haemolymph and other biological matrices likewise do not demonstrate that haemolymph-associated degradation limited injection-mediated RNAi in the species examined.

Injection bypasses salivary, digestive, and gut-epithelial exposures but does not protect dsRNA from potential degradation or other extracellular losses within the haemocoel. Nevertheless, detecting haemolymph-associated degradation does not establish its quantitative contribution to target-transcript depletion, tissue distribution, or the duration of RNAi following injection. It also does not constitute evidence that haemolymph degradation limits systemic RNAi, because systemic movement, tissue exposure, cellular uptake, and downstream silencing were not experimentally linked to the observed ex vivo activity.

Current evidence is largely restricted to selected Pentatomidae and Miridae and is derived predominantly from ex vivo assays [9,13,14,15,16]. Haemolymph-associated dsRNA degradation should therefore be described as a directly demonstrated process in the tested species and experimental matrices, whereas its functional importance in vivo remains unresolved. Determining this importance will require quantitative analysis of dsRNA persistence and integrity in haemolymph in vivo, combined with measurements of tissue exposure, systemic distribution, and the magnitude and duration of target-transcript depletion.

## 4. Physical and Physiological Interfaces and Constraints

Persistence of dsRNA in extracellular fluids is necessary but insufficient to establish that an intact silencing trigger reaches the cells in which the target transcript is expressed. Following oral delivery, dsRNA must reach and traverse the gut interface; after entry into the haemocoel, its functional availability may vary among tissues. Direct measurements of tissue access and dsRNA biodistribution remain limited in Heteroptera, with most available studies providing functional evidence of silencing beyond the administration site rather than quantitative tracing of intact dsRNA or its derivatives [21,22,23,24]. Interpretation therefore requires a distinction among the anatomical presence of a potentially restrictive interface, functional evidence that a silencing signal reaches a particular tissue, and quantitative demonstration of the amount, integrity, and molecular form of dsRNA or its derivatives distributed among tissues.

This section applies that distinction to four questions: whether midgut architecture restricts dsRNA passage, whether cuticular passage limits topical delivery, whether a silencing signal can elicit RNAi effects in internal tissues, and how developmental timing modifies the observed response. Anatomical studies have established the presence and organisation of the PMM in several heteropteran taxa [25,26,27,28,29]. Separately, target-transcript depletion or associated effects in tissues distant from the administration site, together with maternal or systemic responses, demonstrate functional access to target tissues in particular species and experimental contexts [21,22,23,24]. Such observations do not establish the PMM as a causal barrier or define the route, quantity, integrity, and molecular form of the transmitted silencing signal. Conversely, detection of tissue-associated dsRNA or a corresponding fluorescent signal would demonstrate distribution but would not, without additional evidence, establish productive cellular uptake or engagement of the RNAi pathway.

### 4.1. Midgut Architecture: A Candidate Interface Rather than a Demonstrated Barrier

The midguts of many hemipterans lack the peritrophic matrix found in numerous other insects and instead possess a PMM associated with the apical microvilli. The occurrence and ultrastructure of the PMM have been documented across paraneopteran lineages, including Heteroptera [25,26,27]. In *Eurygaster integriceps* Puton, 1881, biochemical fractionation distinguished microvillar and perimicrovillar membrane domains, and PMM formation varied in response to feeding [28,29]. These studies establish the presence of a specialised, physiologically dynamic gut interface, but they did not examine dsRNA transport.

The PMM’s position between the gut lumen and the epithelial surface makes it a plausible candidate for influencing the access of large RNA molecules to epithelial cells. Nevertheless, no study in Heteroptera has quantified dsRNA permeability across the PMM, visualised the retention of intact dsRNA within the perimicrovillar space, or demonstrated that experimental alteration of PMM integrity affects target-transcript depletion. The PMM should therefore be described as an untested interface rather than a demonstrated barrier to oral RNAi. Weak oral responses cannot be attributed to this structure without distinguishing its potential effects from extracellular degradation, limited epithelial passage, and subsequent cellular or tissue-level losses.

The localisation experiments reported by Qiao et al. [20] do not constitute a test of dsRNA passage across the PMM. The fluorescently traced component was chitosan rather than dsRNA, the integrity of the associated dsRNA was not assessed, and the observed signal was not localised relative to the PMM. Consequently, these findings demonstrate the distribution of the labelled carrier under the tested conditions but do not establish that intact dsRNA traversed the PMM or reached the gut epithelial cells in a biologically available form.

Direct testing would require time-resolved localisation of labelled dsRNA on the luminal and epithelial sides of the PMM, accompanied by an integrity-sensitive assay capable of distinguishing intact dsRNA from labelled degradation products. This analysis should be combined with an intervention that alters PMM organisation, followed by measurement of the resulting target-transcript depletion. Recovery and integrity analysis of dsRNA from the gut epithelium and haemolymph would further help distinguish retention at the interface from successful passage. Until such evidence becomes available, PMM-mediated restriction remains a diagnostic hypothesis in Heteroptera.

### 4.2. Cuticular Passage: Route-Level Evidence Without a Resolved Mechanism

Topical delivery introduces a cuticular interface distinct from the midgut-associated interfaces encountered during feeding. In *A. lucorum*, direct topical application of SPc-complexed Cy3-labelled dsRNA produced greater fluorescence intensity in washed whole nymphs and in longitudinal sections than application of naked labelled dsRNA [18]. RT-qPCR-based estimation also indicated greater dsGFP abundance in whole insects following direct topical application of the SPc formulation [18]. Directly applied SPc-complexed target dsRNA produced target-transcript depletion and increased nymphal mortality, providing functional evidence that the silencing trigger reached responsive cells under the tested conditions [18].

These findings support carrier-assisted topical delivery at the route level but do not quantify the amount of intact dsRNA that crossed the cuticle or establish its subsequent tissue and cellular localisation. Fluorescence and whole-insect RT-qPCR measurements cannot distinguish intact internalised dsRNA from labelled or amplifiable degradation products, nor can they completely exclude residual surface-associated material. Moreover, SPc may affect several processes simultaneously, including surface spreading, retention, extracellular stability, passage across external interfaces, and subsequent cellular delivery. The results are therefore consistent with functional passage following direct topical administration but do not resolve the physical entry route or identify the process responsible for the improved response [18].

In the spraying experiment, the formulation was applied to bean plants infested with *A. lucorum* nymphs; target-transcript depletion and increased mortality were subsequently observed [18]. This design constitutes a mixed or unresolved exposure regime because direct cuticular contact, ingestion from treated plant material, and other routes of acquisition were not separated experimentally [18]. The spraying results consequently demonstrate formulation efficacy under the tested exposure conditions but do not provide specific evidence that cuticular passage was the operative delivery route [18].

Reduced cuticular permeability has therefore not been established as a limiting mechanism in *A. lucorum* or other Heteroptera. Direct assessment would require rigorous removal and quantification of surface-associated material, integrity-sensitive recovery of internal dsRNA, depth- and tissue-resolved localisation, and an intervention that selectively alters cuticular passage while leaving extracellular protection and cellular uptake unaffected.

### 4.3. Functional Tissue Access and Unresolved Molecular Distribution

Evidence from Heteroptera shows that an administered silencing trigger can produce target-specific effects in tissues distant from its point of entry. In *R. prolixus*, ingested dsRNA targeting *nitrophorin 2* reduced the corresponding transcript level in the salivary glands by 42 ± 10%, compared with 75 ± 14% after injection [21]. In *N. viridula*, injection of dsRNA targeting *white* produced phenotypes in several adult tissues and reduced the target-transcript level by more than 99% in eggs laid by treated females, demonstrating a maternal RNAi effect under the tested conditions [24]. Injection-mediated silencing of *vitellogenin* in *C. lectularius* also affected the ovary and fat body and strongly suppressed egg production [23]. These outcomes demonstrate functional completion of the processes required to produce target-specific effects in particular tissues or, in the case of maternal RNAi, in the progeny. They do not identify the molecular species reaching each responsive site, establish that intact dsRNA itself was distributed to these sites, or quantify its tissue-level bioavailability.

Functional access is not necessarily uniform. Following abdominal injection of sequence-matched ds*RpIAP* in *R. prolixus*, Finetti et al. [22] detected strong target-transcript depletion in the fat body sampled near the administration site but not in the head. This tissue-resolved result demonstrates that RNAi output can vary among tissues within the same insect. Because the distribution of intact dsRNA, its degradation products, and siRNAs was not measured, the absence of detectable transcript depletion in the head cannot distinguish limited transport from insufficient cellular uptake, differences in target-transcript kinetics, or inefficient intracellular processing. Conversely, successful transcript depletion in a particular tissue demonstrates that the processes required to generate a local RNAi response were completed under the tested conditions but does not establish equivalent exposure or responsiveness in other organs.

In *A. lucorum*, fluorescence associated with a labelled chitosan carrier was detected within the gut and haemocoel after oral administration of chitosan-complexed dsRNA [20]. Because the fluorescent label was attached to chitosan rather than dsRNA, these observations trace the carrier but do not demonstrate the distribution or integrity of the associated dsRNA. The signal therefore does not establish the tissue-level bioavailability of biologically active dsRNA. The subsequent target-transcript depletion demonstrates functional completion of the overall delivery sequence under the tested conditions, but it does not directly link the observed carrier-associated fluorescence to intact or biologically active dsRNA.

The available evidence therefore demonstrates functional effects in defined tissues and provides limited spatial observations of carrier distribution in particular species and experimental contexts [20,21,22,23,24]. It does not resolve the quantitative tissue distribution, integrity, or molecular form of the biologically active silencing trigger. Whole-body transcript measurements, organism-level phenotypes, and carrier-associated fluorescence cannot, on their own, address this uncertainty. Time-resolved recovery of labelled dsRNA and analysis of its integrity, combined with tissue-specific quantification of dsRNA, siRNAs, and target-transcript depletion, are required to distinguish molecular distribution from local cellular responsiveness.

### 4.4. Developmental Timing and Physiological Context

Developmental stage can alter the observable consequences of gene silencing without necessarily changing the intrinsic efficiency of the RNAi machinery. In *N. viridula*, injection of dsRNA targeting *white* produced substantial depletion of the target transcript in fifth-instar nymphs and newly emerged adults, whereas the development of adult pigmentation phenotypes depended on the timing of treatment relative to the imaginal moult [24]. This experiment demonstrates a developmental window for phenotypic manifestation; it does not establish that dsRNA uptake or intracellular processing was intrinsically less efficient at either stage.

Other sources of variation are harder to interpret because the target gene, dose, delivery route, sampling time, and biological context often vary simultaneously across studies. Studies in *P. stali* and tissue-resolved injection experiments in *R. prolixus* demonstrate that molecular and organism-level outcomes depend on the specific target–route–dose–time combination examined [22,32]. However, these studies did not isolate nutritional state, reproductive status, or environmental conditions as causal determinants of RNAi performance. These variables should therefore be regarded as insufficiently tested sources of context dependence rather than as demonstrated constraints on RNAi in Heteroptera.

Accordingly, developmental stage, sex, feeding state, and reproductive status should be recorded as explicit experimental-design and reporting variables. Target-transcript depletion, protein-level effects, and organismal phenotypes should be assessed separately and at biologically appropriate time points. In summary, the PMM represents an untested candidate interface, functional effects in different tissues have been documented but are not necessarily uniform, and developmental timing can alter phenotypic manifestation without demonstrating a corresponding difference in intrinsic RNAi efficiency. These conclusions identify testable sources of context dependence without presenting them as universal causes of RNAi inefficiency.

## 5. Cellular Steps and Candidate Constraints

Cellular entry is required for exogenous dsRNA to engage the cytoplasmic siRNA pathway, but an obligatory step is not necessarily a demonstrated cause of RNAi failure. Three questions must therefore be distinguished: whether dsRNA can enter heteropteran cells, whether endocytic uptake results in productive cytoplasmic access, and whether a functional silencing effect can occur in tissues distant from the site of administration. Current evidence addresses these questions at markedly different levels of resolution.

In Heteroptera, target-transcript depletion following haemocoelic injection [21,24] and, in selected experimental systems, oral delivery [14,21] provide functional evidence that the steps required for productive intracellular RNAi can be completed. In *A. lucorum*, target-transcript depletion was also obtained following direct topical application of SPc-complexed dsRNA [18]. By contrast, spraying SPc-complexed dsRNA onto plants infested with *A. lucorum* nymphs constituted a mixed or unresolved exposure regime because direct cuticular contact, ingestion of dsRNA from treated plant material, and other routes of acquisition were not separated experimentally [18]. Its effects therefore cannot be assigned specifically to either topical or oral delivery. Collectively, these findings demonstrate that the delivery and intracellular processing steps required for target-transcript depletion can be completed under the respective experimental conditions. They do not identify the cellular uptake pathway involved or quantify its efficiency.

Short RNA products of a size consistent with siRNAs have also been detected after injection [15]. However, classifying them by size alone does not establish their molecular identity, loading into Argonaute, or functional contribution to target silencing. Similarly, transcript depletion following any route of administration demonstrates productive completion of the overall delivery sequence but does not resolve cellular entry, endosomal release, or intracellular processing as separate steps.

Endosomal escape has not been examined directly in Heteroptera. Systemic and maternal RNAi effects have been functionally documented [21,24], but these outcomes do not identify the transported molecular species or distinguish between extracellular distribution of the administered trigger and cell-to-cell propagation of a silencing signal. Cellular processes should consequently be classified as demonstrated functions, candidate mechanisms, or unresolved evidence gaps rather than grouped as established barriers.

### 5.1. Cellular Uptake: Functional Evidence and Unresolved Mechanism

Mechanistic evidence from other insects shows that dsRNA uptake can involve defined endocytic pathways. In *Drosophila melanogaster* S2 cells, disruption of endocytic components reduced dsRNA internalisation and RNAi activity, and class C scavenger receptors were shown to contribute to dsRNA uptake [39,40]. These experiments establish endocytosis as a productive uptake mechanism in this cellular model. However, they do not demonstrate that the same components mediate dsRNA uptake in Heteroptera or that their activity limits RNAi performance in vivo.

Direct pathway-level tests have not been reported for heteropteran cells. Transcriptomic analyses of *E. heros* and *Piezodorus guildinii* (Westwood, 1837) identified candidate components associated with endocytosis but did not detect SID-like homologues [9,30]. These annotations provide testable candidates for uptake studies, whereas failure to detect a homologue in a transcriptome does not establish its genomic absence or indicate functional failure of dsRNA uptake.

In *A. lucorum*, Qiao et al. [20] detected fluorescence associated with orally administered complexes containing fluorescently labelled chitosan and dsRNA. However, the chitosan carrier, rather than the dsRNA, was labelled. The study did not assess co-localisation with plasma membrane or endocytic markers, and it did not exclude dissociation of the fluorescent label, carrier degradation, or carrier fragmentation. Carrier-associated fluorescence therefore does not demonstrate dsRNA internalisation or establish that intact dsRNA entered the observed cells. The subsequent target-transcript depletion supports successful completion of the steps required for productive RNAi under the tested conditions but does not directly link the fluorescent signal to biologically active dsRNA or resolve cellular uptake as an individual step.

At the organismal level, detection of short RNA products of a size consistent with siRNAs after injection in several heteropterans provides evidence consistent with cellular entry and intracellular processing of at least part of the administered dsRNA [15]. However, size-based detection alone does not confirm that these products were target-specific siRNAs or that they were incorporated into functional RNA-induced silencing complexes. Likewise, oral depletion of the *HhCHC* transcript following dsDNA-mediated protection of dsRNA in *H. halys* demonstrates productive completion of the delivery chain under the tested conditions, but cellular uptake was not measured directly [14].

Productive entry is therefore functionally supported in particular heteropteran species and experimental systems, but neither the mechanism nor the efficiency of cellular uptake has been measured directly. Reduced uptake should remain an unresolved candidate explanation rather than a probable general constraint in Heteroptera. A causal test would require quantitative internalisation assays in defined primary cells or tissues, experimental exclusion or removal of surface-bound dsRNA, subcellular localisation relative to the plasma membrane, and perturbation of candidate endocytic components. These experiments should be followed by measurement of cytoplasmic dsRNA, target-specific siRNAs, and target-transcript depletion. Whole-body target-transcript measurements by reverse-transcription quantitative PCR (RT-qPCR), mortality assays, carrier-associated fluorescence, or improved transcript depletion following dsRNA stabilisation cannot, on their own, identify cellular entry as the limiting step.

### 5.2. Endosomal Trafficking as an Unresolved Comparative Hypothesis

Endocytic uptake does not necessarily provide access to the cytoplasm. In *Spodoptera frugiperda* (J.E. Smith, 1797), labelled dsRNA accumulated within endosomal compartments, whereas lipid-based interventions altered its intracellular distribution and enhanced RNAi activity [41,42]. These non-heteropteran findings constitute class D comparative evidence: they provide a mechanistic model and an experimental framework for investigating intracellular trafficking but do not establish that endosomal entrapment occurs or limits RNAi performance in Heteroptera.

Qiao et al. [20] proposed proton-sponge-mediated endosomal escape as a model for the action of chitosan in *A. lucorum*. However, neither endosomal localisation nor dsRNA release into the cytoplasm was examined. The mechanistic scheme presented by the authors should therefore be regarded as a proposed explanation for the observed formulation effects rather than as direct evidence of endosomal escape. Similarly, the improved delivery and RNAi outcomes obtained with SPc-complexed dsRNA do not demonstrate SPc-mediated endosomal escape because subcellular localisation and cytoplasmic release were not assessed [18].

No study in the available heteropteran evidence base has directly assessed the co-localisation of dsRNA with endosomal or lysosomal markers, quantified its release into the cytoplasm, or selectively manipulated endosomal escape while controlling for extracellular stability and total cellular uptake. Improvements associated with liposomes, EDTA, dsDNA co-formulation, chitosan, or SPc cannot resolve this question because these interventions may alter extracellular persistence, exposure, cellular uptake, intracellular trafficking, or several of these processes simultaneously [11,14,18,20]. Accordingly, partial target-transcript depletion, the absence of mortality, or the use of a protective or carrier-based formulation should not be interpreted as evidence either for or against endosomal retention.

Testing this hypothesis requires time-resolved subcellular localisation using an approach that can distinguish intact dsRNA from labelled degradation products. Such analysis should be combined with an intervention that selectively modifies endosomal trafficking or escape, while extracellular stability and total cellular uptake are quantified independently. Detection of intact dsRNA in the cytoplasm, followed by measurement of target-specific siRNAs and target-transcript depletion, would be required to determine whether endosomal retention is a functionally limiting step.

### 5.3. Functional Systemic Effects and Unresolved Signal Movement

Systemic RNAi can be defined operationally as sequence-specific silencing of target genes in cells or tissues beyond those initially exposed to the silencing trigger. This definition describes a spatially non-local functional outcome but does not specify which molecular species transmits the silencing information. Selected studies in Heteroptera support systemic or maternal effects under particular experimental conditions [21,24], as discussed in Section 4.3. Maternal RNAi represents a related transgenerational outcome but likewise does not identify the molecular form transferred to reproductive tissues or progeny.

The available evidence does not distinguish between the movement of intact long dsRNA and the transport of primary siRNAs or other sequence-specific signals. Consequently, transcript depletion in a distant tissue cannot, on its own, establish that intact dsRNA reached that tissue, whereas strong whole-body depletion does not demonstrate homogeneous systemic distribution. A canonical RNA-dependent RNA polymerase system that generates secondary siRNAs has not been demonstrated in insects [43,44]. Secondary amplification should therefore not be invoked to explain sustained or non-local effects in Heteroptera. In the absence of such amplification, persistence and intertissue distribution of the administered dsRNA, movement of its primary processing products, and repeated uptake by responsive cells may explain these effects, but their relative contributions remain undetermined.

Mechanistic resolution will require time-resolved transfer assays in which haemolymph or tissue-derived material from treated insects is tested for sequence-specific silencing activity in untreated recipients. These experiments should be combined with integrity-sensitive recovery and molecular characterisation of long dsRNA, together with tissue-resolved small-RNA profiling capable of distinguishing primary siRNAs from other RNA species. Parallel measurement of target-transcript depletion in the same tissues and at corresponding time points would permit the spatial distribution of candidate signals to be correlated with functional RNAi output.

Systemic and maternal effects should therefore be described as functionally demonstrated in selected heteropteran systems, whereas the route of signal movement and the identity, integrity, and relative contribution of the mobile molecular species remain unresolved [21,24]. Overall, the evidence reviewed in Section 5 supports the successful completion of the steps required for productive intracellular RNAi and non-local silencing in specific experimental systems, but it does not establish reduced cellular uptake or endosomal retention as general constraints in Heteroptera. The following section examines whether inefficiency in downstream intracellular processing is supported as a functionally limiting mechanism.

## 6. Intracellular RNAi Machinery: Functional Capacity and Unresolved Constraints

Productive RNAi requires cytoplasmic dsRNA to be processed into siRNAs, followed by loading of a guide strand into an Argonaute-containing RISC and sequence-specific depletion of the target transcript [2,43]. These are obligatory intracellular steps, but they should not be regarded as demonstrated constraints unless the relevant molecular intermediate has been measured and causally linked to a weak RNAi response.

The available evidence does not support a general absence of intracellular RNAi competence in Heteroptera. Transcripts encoding core components of the siRNA pathway have been identified in *E. heros* and *P. guildinii* [9,30]. In addition, short RNA products of a size consistent with siRNAs have been detected after dsRNA injection in several heteropterans [15], and haemocoelic delivery has produced target-transcript depletion or phenotypic effects across multiple families [10,13,21,23,24,31,32,33,34,35,36,37]. Collectively, these observations support the presence of candidate pathway components and, where target-transcript depletion was confirmed, demonstrate that the intracellular processes required to generate a target-specific RNAi response can be completed in the tested systems. However, size-based detection alone does not confirm the sequence identity of the observed short RNAs or their functional incorporation into RISC. Moreover, the available studies generally do not quantify Dicer-mediated processing, Argonaute loading, or target-RNA cleavage, which remain largely uncharacterised in Heteroptera. The following assessment therefore distinguishes the presence of candidate pathway components, functional evidence of downstream silencing, and unresolved intracellular mechanisms.

### 6.1. Core Components of the Insect siRNA Pathway

In the canonical exogenous siRNA pathway, Dicer-2 cleaves long cytoplasmic dsRNA into siRNA duplexes. A guide strand is subsequently retained by Argonaute 2 (AGO2) within the RISC, which recognises complementary target RNA and mediates its cleavage. In well-characterised insect systems, dsRNA-binding cofactors, including R2D2 and Loquacious isoforms, contribute to dsRNA processing, strand selection, or RISC loading [2,43]. Inefficiency at any of these steps is mechanistically possible, but the pathway architecture alone does not identify which, if any, step limits an observed heteropteran response.

The most direct heteropteran evidence combines pathway annotation with downstream functional outcomes. In *E. heros*, transcripts corresponding to *Dcr2*, *Ago2*, and *R2D2* were identified, injection of dsRNA targeting *vATPase A* reduced the level of the corresponding transcript, and *Dcr2* and *Ago2* transcript levels increased following treatment [9]. In *P. guildinii*, injection of dsRNA targeting *vATPase A* produced 64–84% target-transcript depletion over 24–72 h and cumulative mortality of 51.6% by day 14 [30]. These studies demonstrate that intracellular silencing processes can support substantial target-transcript depletion under the tested conditions. However, treatment-associated changes in the expression of pathway-component genes do not, in themselves, establish the abundance or activity of the encoded proteins, nor do they identify a functionally limiting component. Likewise, detection of short RNA products of a size consistent with siRNAs after injection provides evidence consistent with dsRNA processing, but these products were not sequence-verified as target-derived siRNAs, nor was their loading into active RISC demonstrated [15].

Accordingly, the available evidence supports the functional capacity of the core siRNA pathway in the examined heteropteran systems [9,15,30], whereas the efficiency of its individual steps remains unresolved. Direct attribution of a weak RNAi response to inefficient intracellular processing would require target-specific small-RNA profiling, biochemical characterisation of Dicer-2 products, recovery of target-derived siRNAs from AGO2-containing complexes, or functional perturbation of candidate pathway components followed by matched measurements of siRNA abundance and target-RNA cleavage.

### 6.2. Conservation and Variation in RNAi-Related Genes in Heteroptera

Transcriptomic analyses of *E. heros* and *P. guildinii* identified transcripts encoding candidate Dicer, Argonaute, and dsRNA-binding proteins, while comparative surveys revealed conserved domains in several RNAi-associated proteins across hemipteran species [9,15,30]. These findings support the presence and evolutionary conservation of major components of the molecular framework underlying RNAi. However, sequence recovery does not demonstrate equivalent biological function. In particular, it does not establish tissue-specific expression, protein abundance, responsiveness to treatment, catalytic activity, or the coordinated assembly of a functional RNA-induced silencing complex.

Reported differences in gene copy number, domain architecture, recovered isoforms, or expression patterns generate testable hypotheses concerning variation in RNAi pathway output [45]. Their interpretation is constrained by the predominance of transcriptomic datasets rather than complete, consistently annotated genomes. An apparent absence from a transcriptome may reflect incomplete assembly, restricted or low-level expression, the tissues or developmental stages sampled, or difficulties in assigning paralogues to different small-RNA pathways. Taxonomic coverage is also uneven: Pentatomidae account for the most detailed molecular datasets, whereas many heteropteran families remain poorly characterised. Gene-content patterns should therefore neither be generalised across Heteroptera nor presented as demonstrated causes of variation in RNAi performance.

Comparisons among delivery routes reinforce this distinction. Substantial target-transcript depletion after injection demonstrates that the available cellular machinery can support RNAi under the tested conditions when luminal exposure and passage across the gut interface are bypassed [9,13,23,24,30,31,32]. When weak responses occur after oral or direct topical delivery, they cannot be attributed specifically to deficiencies in intracellular pathway components because productive cytoplasmic exposure has not been established [10,11,12,14,32,33]. Responses after direct topical application or mixed-route spraying likewise do not, on their own, resolve intracellular pathway efficiency because the amount of biologically active dsRNA reaching the cytoplasm was not quantified [18]. Conversely, a weak response after injection increases the relevance of downstream cellular explanations but does not identify intracellular processing as the limiting step, because extracellular stability in the haemolymph, tissue distribution, cellular uptake, administered dose, target-transcript abundance, and response timing may also influence the observed outcome [22]. Comparative genomics becomes mechanistically informative only when candidate gene-content or sequence variation is linked to tissue-resolved expression, target-specific small-RNA production, and functional perturbation of the relevant pathway component.

### 6.3. Absence of a Canonical RNA-Dependent RNA Polymerase System and Consequences for Signal Amplification

No canonical RNA-dependent RNA polymerase (RdRP) system equivalent to the secondary-siRNA amplification pathways found in plants and nematodes has been identified in insects, and canonical RdRP-mediated secondary amplification has not been demonstrated experimentally [2,43,44]. Current molecular and comparative evidence therefore strongly supports the absence of such a canonical amplification system. This represents a structural difference among RNAi systems rather than evidence that heteropterans cannot produce systemic or persistent RNAi responses. Functional systemic and maternal effects have been documented in selected species, demonstrating that such outcomes can occur without a known canonical RdRP-mediated amplification pathway [21,24].

On biological grounds, the absence of demonstrated secondary amplification would be expected to increase dependence on primary exposure and the subsequent fate of the administered trigger: the amount of dsRNA reaching the cytoplasm, its processing into primary siRNAs, and the persistence or renewed cellular availability of the delivered trigger or its primary processing products [2,43,44]. This is a mechanistic interpretation rather than a directly measured property of RNAi in Heteroptera. Although it is consistent with the frequently stronger responses observed after injection and with transient or threshold-dependent outcomes, the contribution of primary exposure to the magnitude and duration of silencing has not been directly quantified in heteropterans. A short-lived reduction in target-transcript abundance should therefore not automatically be attributed to deficient Dicer or RISC activity. Depletion of the administered trigger, turnover of responsive cells, or restoration of target-gene transcription could produce such transient molecular patterns, whereas persistence of the encoded protein could delay or attenuate the phenotypic effect despite transcript depletion.

The practical consequence is diagnostic rather than taxonomic. When a phenotype requires sustained target suppression, a single RT-qPCR measurement of transcript abundance cannot determine whether primary siRNA production was insufficient or whether an initially detectable response declined before achieving the magnitude or duration of suppression required to affect the phenotype. Time-resolved quantification and integrity analysis of dsRNA, sequence-specific measurement of target-derived primary siRNAs, and matched assessment of target-transcript abundance, protein abundance, and phenotype are required to distinguish loss of the silencing trigger from inefficient intracellular processing. The conclusion that canonical RdRP-mediated secondary amplification is absent is well supported, whereas the inferred greater dependence on primary dsRNA exposure remains a biologically justified but untested interpretation in Heteroptera.

### 6.4. Functional Evidence and Diagnostic Limits After dsRNA Delivery

Most heteropteran studies infer intracellular RNAi competence from downstream endpoints rather than directly measuring Dicer-2-mediated processing, AGO2 loading, RISC activity, or target-RNA cleavage. Target-transcript depletion following experimental dsRNA delivery has been documented in Pentatomidae [9,10,11,12,13,14,24,30,31,32], Cimicidae [23,33,34], and Reduviidae [21,22]. By contrast, studies in *O. fasciatus* have largely relied on phenotype-based evidence from developmental gene-function experiments [35,36,37]. These results support sequence-specific functional effects after dsRNA treatment in this species but should not be cited as direct evidence of target-transcript depletion in Lygaeidae or of any individual intracellular step.

In Miridae, transcript-level effects have been reported in three distinct experimental contexts in *A. lucorum*: feeding on transgenic plants expressing target-specific dsRNA [19], feeding on beans treated with chitosan-complexed dsRNA [20], and direct topical application or plant spraying of SPc-complexed dsRNA [18]. The spraying experiment represents a mixed exposure regime because direct cuticular contact and ingestion of dsRNA from treated plant material were not separated. Collectively, these outcomes support successful completion of the sequence of delivery and intracellular processes required to produce target-transcript depletion under the respective conditions. They do not constitute direct evidence of Dicer-2 processing, AGO2 loading, RISC activity, or target-RNA cleavage. Moreover, the carrier-assisted treatments do not identify which extracellular, physical, cellular, or intracellular process the formulation altered. Conversely, a weak response after injection is compatible with an intracellular constraint but does not distinguish it from extracellular degradation, heterogeneous tissue distribution, insufficient cytoplasmic exposure, target-transcript abundance, or inappropriate sampling time.

Tissue-resolved results illustrate this diagnostic boundary. Following abdominal injection in *R. prolixus*, depletion of the *RpIAP* transcript was substantial in the fat body sampled near the injection site but was not detected in the head, and survival was reduced [22]. This finding demonstrates a spatially heterogeneous functional response. It does not establish either that dsRNA failed to reach the head or that intracellular processing was deficient in that tissue because neither dsRNA biodistribution nor target-derived siRNAs was measured. Conversely, whole-body transcript measurements may obscure substantial depletion within a small responsive tissue by diluting its contribution to the aggregate signal, even when the local effect is biologically decisive.

Target-transcript depletion and organismal phenotype must also be treated as distinct endpoints. In *H. halys*, *CAT* transcript abundance was strongly reduced after injection, with no significant effect on survival [31]. For *HhCHC*, an orally delivered dsDNA-protected formulation produced target-transcript depletion without mortality, whereas injection resulted in greater transcript depletion and substantial mortality [14]. In *N. viridula*, depletion of the *white* transcript was associated with pigmentation phenotypes whose manifestation depended on the timing of treatment relative to development [24]. These findings demonstrate that molecular and organismal outcomes need not correspond directly. Their relationship may depend on target function, tissue specificity, the magnitude and duration of transcript depletion, protein persistence, developmental timing, and the organismal endpoint examined. Transcript–phenotype discordance does not, on its own, implicate deficient Dicer-2 processing, RISC activity, or AGO2 function.

The converse mismatch warrants equal caution. After topical treatment of *H. halys*, reduced survival was reported for one target–concentration combination with no significant target-transcript depletion at the sampled time points [22]. Mortality alone cannot demonstrate sequence-specific RNAi or substitute for an appropriate molecular endpoint. Each target–dose–time combination should therefore be assessed using temporally aligned measurements of transcript or protein depletion and a biologically relevant phenotype. This distinction separates RNAi efficacy from insecticidal efficacy and reduces both false-negative and false-positive mechanistic interpretations.

Direct attribution of a weak response to an intracellular constraint requires evidence linking cytoplasmic exposure to pathway-specific intermediates and to downstream target depletion. Without such evidence, strong transcript-level responses support successful completion of the steps required for productive intracellular RNAi, whereas weak or discordant outcomes indicate that the limiting stage within the delivery–response sequence remains unresolved rather than demonstrating a molecular bottleneck. Phenotype-based evidence from *O. fasciatus* supports functional gene perturbation but provides less mechanistic resolution than matched measurements of target-transcript depletion and pathway-specific intermediates. Detailed diagnostic designs are considered in Section 8.2.

Overall, transcript-level evidence supports productive RNAi responses in multiple heteropteran families and exposure contexts, while phenotype-based evidence extends the functional evidence base without directly resolving intracellular processing. The available findings do not establish inefficient Dicer-2 processing, RISC loading, or AGO2 activity as general constraints in Heteroptera. The resulting conclusion is necessarily asymmetric: successful downstream outcomes support intracellular RNAi competence under particular experimental conditions, whereas variation within the pathway remains a mechanistically plausible but unresolved contributor to response heterogeneity. Section 7 integrates this uncertainty with the more directly supported extracellular and delivery-route-dependent losses without assigning equivalent evidential weight to all proposed constraints.

## 7. Evidence-Constrained Multilevel Model of RNAi Responsiveness in Heteroptera

RNAi responsiveness in Heteroptera reflects interactions among extracellular, physiological, cellular, and intracellular processes, as well as the subsequent conversion of molecular silencing into an organismal phenotype. However, the evidence supporting these processes is uneven. Degradation of dsRNA in native biological fluids has been directly demonstrated in selected heteropterans, including rapid degradation in saliva from *L. lineolaris* [16] and degradation in midgut fluid from *A. lucorum* [17,18], alongside corresponding evidence from other species [9,10,11,12,13,14,15]. Zhang et al. [17] also demonstrated dsRNA degradation by purified recombinant AldsRNase. This purified-enzyme activity establishes the protein’s catalytic capacity under the tested in vitro conditions but is distinct from degradation measured in a native biological fluid. Both evidence types must also be distinguished from formulation-dependent dsRNA protection and from experiments causally linking an intervention to improved RNAi output.

Functional systemic and maternal effects have been demonstrated only in particular species and experimental contexts [21,24]. Nevertheless, downstream target-transcript depletion supports successful completion of the delivery–response sequence in multiple heteropterans. In *A. lucorum*, such functional completion has been observed following plant-mediated exposure, chitosan-assisted oral feeding, direct topical application of SPc-complexed dsRNA, and plant spraying under a mixed or unresolved exposure regime [18,19,20]. These outcomes do not directly measure cellular uptake, endosomal escape, Dicer-2-mediated processing, AGO2 loading, or RISC activity. In particular, the protective and functional effects of chitosan do not establish that the carrier increased dsRNA internalisation or mediated endosomal escape. Although gut-associated structures have been anatomically characterised [25,26,27,28,29], their effects on dsRNA passage remain unresolved; limited cellular uptake and endosomal retention likewise remain untested as limiting mechanisms in Heteroptera.

**Figure 1:**
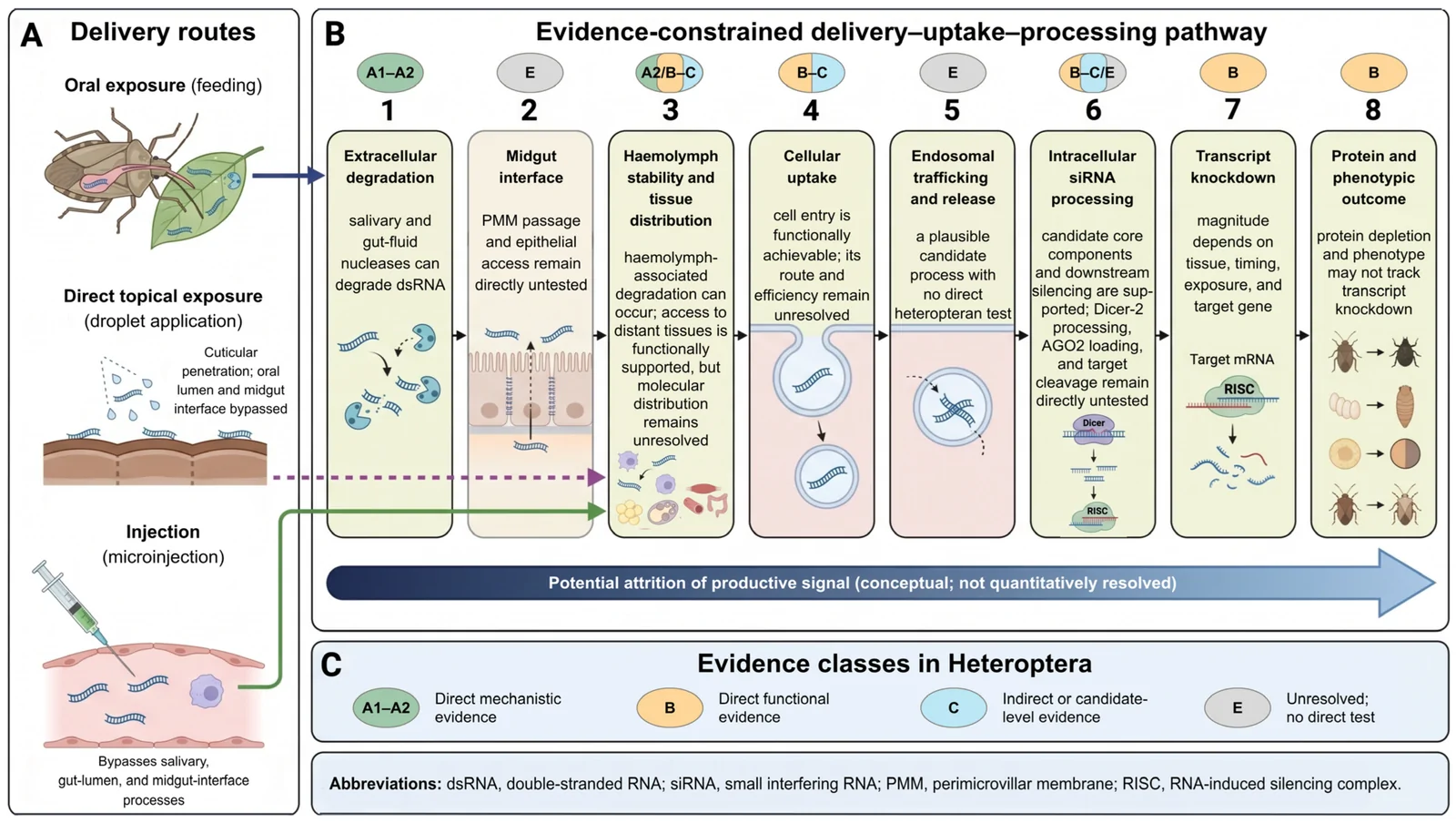
Evidence-constrained multilevel model of RNAi responsiveness in Heteroptera. (**A**) Delivery routes introduce dsRNA at different points within the pathway. Oral exposure subjects dsRNA to salivary and gut-fluid environments and requires passage across the midgut interface. Topical exposure requires cuticular penetration but bypasses oral compartments. The dashed purple arrow denotes the route-level inference that topically applied dsRNA reaches internal compartments, while indicating that the physical entry route and extent of cuticular passage remain unresolved. Microinjection introduces dsRNA directly into the haemocoel, bypassing salivary, gut-fluid, and midgut-interface processes, but not haemolymph-associated degradation, tissue distribution, cellular entry, or intracellular processing. (**B**) The model extends from extracellular exposure through the midgut interface, haemolymph stability and tissue distribution, cellular uptake, endosomal trafficking and cytoplasmic release, and intracellular processing to target-transcript depletion, protein depletion, and organismal phenotype. The broad arrow represents potential attrition of the productive silencing signal as an operational concept. Because signal retention has not been quantified across the complete pathway, the model should not be interpreted as a demonstrated linear or multiplicative kinetic process. (**C**) Evidence classes refer exclusively to evidence obtained in Heteroptera: A1, direct mechanistic evidence supported by causal perturbation; A2, direct mechanistic observational evidence; B, direct functional evidence that does not identify the responsible step; C, indirect or candidate-level evidence; and E, unresolved owing to the absence of a direct test. Composite markers indicate that distinct subclaims within a process are supported at different evidence levels. Evidence markers describe the degree of support for the indicated process or subclaim and do not imply that it constitutes a demonstrated limiting barrier. Abbreviations: dsRNA, double-stranded RNA; siRNA, small interfering RNA; PMM, perimicrovillar membrane; RISC, RNA-induced silencing complex. Created with BioRender.com.

The resulting model should be interpreted as an evidence-constrained representation of stages at which a biologically active RNAi signal may be lost, diverted, or rendered insufficient, rather than as evidence that every proposed constraint operates in every species. A terminal measurement of target-transcript depletion or phenotype integrates the entire delivery–response sequence and cannot retrospectively identify the limiting process. Figure 1 depicts the relationships among these stages, whereas Table 2 distinguishes their evidence class, demonstrated taxonomic scope, supported interpretation, and appropriate diagnostic readout.

Within this framework, RNAi responsiveness is treated as a property of a defined experimental system rather than as a fixed attribute of a species or taxonomic group. Its interpretation depends on developmental stage, delivery route, exposure conditions, formulation, target tissue, target-gene biology, sampling time, and the selected endpoint. The model consequently organises these dependencies and constrains inference from delivery-route comparisons and intervention studies without imposing a universal hierarchy of limiting processes across Heteroptera.

### 7.1. Evidence-Constrained Attrition of the Silencing Signal

The administered dsRNA dose defines the initial exposure but does not indicate how much biologically active material reaches the cytoplasmic siRNA pathway in the relevant cells. Between administration and target-transcript depletion, the productive signal may be reduced by degradation, distribution away from the target tissue, retention within extracellular or intracellular compartments, non-productive internalisation, or insufficient persistence. Here, attrition denotes an operational reduction in the signal available to produce a sequence-specific response at successive stages. Because the fractions retained at these stages have not been quantified across the complete pathway, the model should not be interpreted as demonstrating a linear or multiplicative kinetic process.

The extracellular evidence comprises several analytically distinct levels. Degradation assays using native saliva, gut fluid, or haemolymph demonstrate loss of dsRNA in the tested biological matrix [9,10,11,12,13,14,15,16,17,18]. Activity of purified recombinant AldsRNase shows that this enzyme can degrade dsRNA under defined in vitro conditions but does not, by itself, quantify its contribution to dsRNA loss or to RNAi performance in vivo [17]. Formulation experiments showing greater preservation of complexed than uncomplexed dsRNA establish formulation-dependent protection under the tested conditions [14,18,20]. Where nuclease suppression or a protective intervention also improves a matched RNAi endpoint, the evidence supports a causal effect of the intervention on RNAi performance [11,12,14]. For multifunctional formulations such as chitosan- or SPc-based complexes, however, any improvement can be attributed only to the formulation as a whole. It cannot be assigned specifically to protection from degradation because exposure, retention, distribution, cuticular passage, cellular entry, or other processes may also have been altered.

Evidence becomes less mechanistically resolved downstream. Systemic and maternal effects demonstrate that non-local responses can occur [21,24], whereas target-transcript depletion after injection in several species [9,15,30] and after plant-mediated, chitosan-assisted oral, direct topical, or mixed exposure in *A. lucorum* [18,19,20] supports the successful completion of the processes required for productive RNAi. Detection of short RNA products of a size consistent with siRNAs provides additional evidence compatible with intracellular processing but does not establish their sequence identity or incorporation into active RISC [15]. Quantitative passage across gut-associated interfaces remains unresolved despite anatomical characterisation of these structures [25,26,27,28,29]. Cellular uptake efficiency, endosomal release, Dicer-2-mediated processing, and AGO2 loading are likewise insufficiently resolved in Heteroptera.

Attrition may also occur between molecular silencing and the organismal phenotype. Target-transcript depletion may be spatially restricted or insufficient in magnitude or duration to reduce the encoded protein below a functionally relevant threshold. For example, substantial depletion of the *CAT* transcript in *H. halys* was not accompanied by significant mortality, whereas protection of *HhCHC* dsRNA enhanced the oral molecular response without reproducing the mortality observed after injection [14,31]. Conversely, a phenotype without a temporally aligned molecular endpoint cannot establish sequence-specific RNAi. The model therefore treats target-transcript depletion, protein depletion, and organismal phenotype as distinct response levels rather than interchangeable measures of efficacy.

This formulation allows an improvement at a single stage, such as increased extracellular stability, to be interpreted without assuming that all downstream processes have also been improved or that the next limiting stage has been identified. Its purpose is to define the mechanistic resolution supported by a given result and to identify the measurements required to distinguish among competing explanations. The corresponding diagnostic framework is developed in Section 8.

### 7.2. Delivery Route as an Experimental Perturbation

Delivery route determines where administered dsRNA enters the multilevel response pathway. Oral exposure involves ingestion, interactions with saliva and the food matrix, persistence within the gut lumen, passage across the midgut interface, and subsequent access to internal tissues. Microinjection introduces dsRNA into the haemocoel and therefore bypasses salivary, gut-luminal, and midgut-interface processes, whereas direct topical application requires passage across the cuticle. These routes expose the trigger to overlapping but non-identical processes and should therefore be treated as experimental perturbations rather than interchangeable methods of administering an equivalent dose [10,11,12,14,18,21,22,32,33].

A strong injection-induced reduction in the target transcript demonstrates that the dsRNA construct can produce sequence-specific silencing when oral-route processes are bypassed. It does not establish uniform tissue distribution, efficient uptake by every relevant cell type, endosomal release, or equivalent intracellular activity across tissues. Accordingly, when injection produces silencing but oral delivery does not, the comparison broadly implicates processes specific to, or intensified by, oral exposure. It cannot distinguish inadequate ingestion, extracellular degradation, restricted epithelial passage, insufficient internal exposure, or interactions among these factors. The pronounced route dependence reported in *P. stali* illustrates this diagnostic localisation without identifying the responsible mechanism [32].

A similar pattern was reported in *L. lineolaris*: target-specific silencing was effective after injection, whereas feeding experiments did not elicit a corresponding response [16]. However, the injection and feeding results originated from separate experiments and were not dose-matched, precluding a controlled quantitative comparison between routes. The demonstrated degradation of dsRNA by *L. lineolaris* saliva is consistent with the absence of an oral response but does not establish salivary degradation as its sole cause. Differences in ingestion, biologically available dose, epithelial passage, and subsequent internal exposure were not independently resolved.

Conversely, a weak response after injection indicates that bypassing the oral compartments was insufficient to produce the expected molecular effect. The unresolved stage may involve haemolymph-associated degradation, tissue distribution, cellular entry, intracellular pathway activity, or experimental and target-dependent factors. Such an outcome does not, by itself, demonstrate deficient cellular uptake, endosomal retention, or impaired RISC activity. Spatially restricted target-transcript depletion after injection in *R. prolixus* further shows that delivery route and whole-body endpoints alone cannot resolve tissue-specific exposure or responsiveness [22].

Successful silencing of the same target via multiple routes provides complementary functional evidence but does not establish equivalent route efficacy. In *A. lucorum*, injection and plant-mediated exposure both silenced the same target [19], supporting productive RNAi under both experimental conditions. The magnitudes of these responses should not be compared because the biologically available doses and resulting internal exposures were unknown and the experiments differed in design. The findings therefore demonstrate target responsiveness across distinct delivery contexts rather than quantitative equivalence between injection and plant-mediated delivery.

Route comparisons are generally constrained by such differences in internal exposure. A nominal injected dose may substantially exceed the amount of intact dsRNA reaching the haemocoel after oral or topical administration and may partially compensate for downstream inefficiencies [2,32,43,44]. In addition, spraying may combine direct contact with ingestion of dsRNA or other exposure from treated plant material. In *A. lucorum*, responses after direct topical application and spraying of formulated dsRNA onto bean plants infested with nymphs therefore cannot be interpreted as a direct comparison of route efficacy because nominal and biologically available exposures were not equivalent and the spraying design did not isolate a single exposure pathway [18]. Injection is informative for determining whether productive silencing can occur after upstream interfaces are bypassed but is not a direct proxy for environmentally relevant delivery. Similarly, improved oral responses after nuclease suppression or dsRNA protection demonstrate that preserving the extracellular signal can influence RNAi outcome without establishing degradation as the only constraint or confirming that all downstream processes are fully effective [11,12,14].

The delivery route is therefore diagnostically informative because it redistributes exposure along the pathway and alters the processes encountered by the RNAi trigger. It can narrow the location of a constraint but cannot identify an individual mechanism without matched exposure and process-specific measurements. The experimental requirements for interpretable route comparisons are addressed in Section 8.1.

### 7.3. Context-Specific Constraint Profiles

A constraint profile is an evidence-qualified description of the processes most likely to limit RNAi in a specified species, developmental stage, delivery route, formulation, target tissue, and endpoint. It characterises a defined experimental system rather than a fixed species trait and should not be inferred from a single dsRNA construct or delivery method. The same species may therefore show substantial target-transcript depletion after injection, an improved response to protected oral dsRNA, and little response to an unprotected trigger because these treatments engage different processes and generate different levels of internal exposure.

The available evidence supports context-specific profiles rather than a universal heteropteran hierarchy of constraints. Extracellular degradation causally contributed to the oral outcome examined in *N. viridula*, whereas formulation studies in *E. heros* and *H. halys* demonstrated improved dsRNA protection or molecular responses without isolating all downstream processes or producing proportional phenotypic effects [11,12,14]. In *A. lucorum*, SPc complexation protected dsRNA in a midgut-fluid assay and supported molecular and phenotypic responses following direct topical application and plant spraying [18]. However, the contribution of protection relative to other formulation- and delivery-related effects was not isolated, and the spraying regime did not distinguish direct contact from ingestion of dsRNA or other exposure through treated plant material. In *P. stali*, contrasting injection and oral outcomes localised the constraint broadly to processes specific to, or intensified by, oral exposure without identifying an individual mechanism [32]. These studies therefore differ in both their inferred constraint profiles and their mechanistic resolution.

The relationship between pathway engagement and phenotype is likewise context-dependent. Developmental timing affected *white*-associated phenotypes in *N. viridula*, whereas spatially restricted target-transcript depletion was observed in *R. prolixus* [22,24]. Target genes differ in expression level and tissue distribution, their encoded proteins differ in persistence and functional redundancy, and the magnitude and duration of depletion required to produce an organismal effect are target-dependent. Transcript abundance, protein abundance, mortality, fecundity, development, and pigmentation therefore represent distinct endpoints and should not be treated as interchangeable indicators of RNAi responsiveness.

Process-level evidence remains disproportionately concentrated in Pentatomidae, although Miridae are also represented by several process-level and functional studies. These include direct salivary degradation and slower haemolymph-associated degradation in *L. lineolaris*, gut-fluid degradation and purified recombinant-nuclease activity in *A. lucorum*, and functional RNAi responses across injection, plant-mediated, chitosan-assisted oral, direct topical, and mixed or unresolved exposure contexts [16,17,18,19,20]. Nevertheless, degradation, biodistribution, cellular uptake, and intracellular processing remain sparsely examined across most heteropteran families. Functional responses in mirids, reduviids, cimicids, pentatomids, and other lineages do not support characterising Heteroptera as generally deficient in RNAi competence, but uneven taxonomic and experimental coverage does not permit families, species, or feeding guilds to be ranked by intrinsic RNAi responsiveness [18,21,23,33,34].

Accordingly, broad classifications such as ‘RNAi-sensitive’ or ‘RNAi-refractory’ should be replaced by descriptions specifying the experimental context and evidential resolution—for example, injection-responsive at the target-transcript level, orally responsive following dsRNA protection, or unresolved because internal exposure was not quantified. This terminology preserves the distinction between an observed response and its mechanistic interpretation. Section 8 defines how to resolve such constraint profiles experimentally.

## 8. Evidence-Informed Experimental Design and Optimisation

The multilevel model has practical value only if it changes how experiments are designed and interpreted. Optimisation should begin by defining the experimental objective and the process that an intervention is intended to alter. Greater target-transcript depletion or a stronger phenotype does not, by itself, establish whether the treatment improved extracellular persistence, internal exposure, tissue access, cellular entry, intracellular processing, or the translation of molecular silencing into a biological effect. Each intervention should therefore be paired with a readout of its proposed proximal effect.

This approach separates target validation from delivery optimisation. Functional genomics experiments ask whether a construct can engage RNAi and whether target perturbation produces the predicted biological consequence under controlled conditions. Applied experiments additionally ask whether a practical delivery route provides sufficient productive exposure in the relevant tissue. These objectives may use the same dsRNA but interrogate different sets of processes and support different inferences. The following framework uses delivery-route comparisons, staged readouts, matched interventions, and biologically aligned endpoints to localise constraints without assigning causality based solely on the final outcome.

### 8.1. Define the Objective and Use Delivery Route Diagnostically

Microinjection serves as an appropriate reference when the primary objective is to determine whether the selected construct and target can produce sequence-specific silencing when oral or cuticular interfaces are bypassed. In *O. fasciatus*, embryonic and postembryonic injection has produced reproducible target-associated developmental phenotypes, providing functional evidence of RNAi-mediated perturbation [35,36,37]. This phenotype-based evidence should be distinguished from injection-induced target-transcript depletion demonstrated in Reduviidae, Cimicidae, Pentatomidae, and Miridae [9,13,19,21,23,24,30,31,32,33,34]. Neither type of evidence establishes uniform biodistribution, equivalent responsiveness across tissues, or field-relevant efficacy.

Oral, plant-mediated, topical, and injection-based treatments should be interpreted separately because they deliver dsRNA via different interfaces. Oral and plant-mediated experiments depend on ingestion and on the amount of intact dsRNA reaching internal compartments. Topical experiments depend on surface exposure and cuticular passage. Direct topical evidence in Heteroptera remains limited and is derived primarily from experiments in *H. halys* and *R. prolixus* [22], *C. lectularius* [33], and SPc-treated *A. lucorum* [18]. Liu et al. [19] additionally demonstrated molecular and phenotypic responses following plant-mediated exposure in *A. lucorum*. A negative or positive result obtained through any practical route cannot, without intermediate measurements, be assigned to an individual delivery or pathway process.

Diagnostic comparisons should retain the same dsRNA construct, developmental stage, principal sampling times, and molecular endpoint across routes. Substantial injection-induced target-transcript depletion accompanied by a weak oral response broadly localises the constraint to processes introduced or intensified by oral exposure but does not distinguish inadequate ingestion, extracellular degradation, restricted epithelial passage, or insufficient internal exposure. Conversely, a weak injection response indicates that bypassing oral or cuticular interfaces was insufficient and does not, on its own, identify a downstream cellular mechanism. The route-dependent response reported in *P. stali* illustrates this diagnostic narrowing without resolving the responsible process [32].

The unmatched comparisons in *L. lineolaris* and *A. lucorum* discussed in Section 7.2 illustrate complementary uses and limitations of route comparisons: divergent outcomes can localise a constraint only broadly, whereas responsiveness through multiple routes supports construct–target competence without demonstrating equivalent biologically available exposure [16,19].

Nominal doses cannot be assumed to represent equivalent biologically available exposure across delivery routes [2,43,44,46]. An injected amount is a defined administered dose, whereas a dietary or plant-associated concentration must be distinguished from the amount ingested and from the intact fraction reaching the haemocoel or target tissue. Similarly, a topical dose must be distinguished from the amount retained on the insect surface, the amount crossing the cuticle, and the amount reaching internal compartments [18,22,33]. Route comparisons should therefore report doses in route-appropriate terms and quantify ingestion, surface retention, dsRNA integrity, and internal exposure, as appropriate. Without these measurements, an apparent route effect may reflect unequal productive exposure rather than a distinct biological constraint.

### 8.2. Apply a Staged Diagnostic Sequence

A diagnostic experiment should proceed from the earliest route-specific process towards the selected biological endpoint. The first level comprises exposure and extracellular persistence. Feeding studies should determine whether dsRNA is ingested and remains intact in the relevant diet, saliva, gut contents, or gut fluid. Injection-based studies may require assessing dsRNA stability in the haemolymph, whereas topical studies require measurements of surface retention and passage beyond the cuticle. Ex vivo degradation assays identify potentially degradative environments, but causal attribution requires perturbation of the proposed process, verification of the resulting change in dsRNA persistence, and a concordant change in a downstream RNAi endpoint. The intervention’s effect on the proposed process must also be distinguishable from its simultaneous effects on formulation, exposure, or delivery [10,11,12,13,14,18].

The available studies show varying levels of evidence. Zhang et al. [17] demonstrated dsRNA degradation and the activity of purified AldsRNase but did not causally test whether this activity limited RNAi. Qiao et al. [20] demonstrated physicochemical and RNase A protection by a chitosan-based formulation but did not test protection in native midgut fluid. Qiao et al. [18] subsequently demonstrated protection in native *A. lucorum* midgut fluid; however, because the formulation could influence several stages of exposure and delivery, its downstream molecular and phenotypic effects cannot be attributed specifically to extracellular protection. Similarly, in *L. lineolaris*, salivary degradation was consistent with the absence of an oral response, but the degradation and RNAi experiments were not causally linked, and the injection and feeding treatments were conducted separately without dose matching [16]. This correspondence supports salivary degradation as a mechanistic hypothesis but does not establish it as the sole cause of oral non-response.

The second level concerns spatial access. Detection of dsRNA in a whole insect does not establish that the intact trigger reached the tissue in which the target gene is expressed and its encoded product performs the relevant function. Tissue-resolved quantification, time-resolved sampling, and imaging of appropriately validated labelled dsRNA can be used to examine distribution. Fluorescence alone should not be equated with intact or biologically active dsRNA because the label may persist after degradation or become spatially separated from the RNA molecule. When the selected phenotype depends on a discrete organ or tissue, tissue-specific measurement of target-transcript abundance by RT-qPCR is more informative than whole-body analysis. Cellular internalisation and cytoplasmic availability require separate assays; neither tissue-level detection nor cellular uptake alone demonstrates productive cytoplasmic delivery.

The third level assesses intracellular pathway engagement and target-transcript depletion. Detection of sequence-verified, target-derived siRNAs would provide strong evidence that administered dsRNA had entered the small-RNA pathway, although it would not independently resolve Dicer-2 activity, AGO2 loading, or target cleavage. By contrast, products identified solely by their apparent size, as in [15], are consistent with dsRNA processing but do not establish their sequence identity or pathway origin. Mechanistic studies should therefore use target-specific small-RNA sequencing or comparably sequence-resolved methods. Target-transcript abundance should be quantified by RT-qPCR at multiple time points and, where biologically justified, in the relevant tissue. This approach distinguishes absent, delayed, transient, and persistent responses and prevents a single time-point result from being interpreted as an intrinsic measure of RNAi responsiveness. Direct measurements are required before inefficiency in Dicer-2 processing, RISC loading, or AGO2-associated activity can be assigned as a limiting process.

The fourth level links target-transcript depletion to protein depletion and organismal phenotype. Reduced transcript abundance does not establish depletion of a stable encoded protein, while phenotype development additionally depends on tissue specificity, physiological compensation, functional thresholds, and the biological window of observation. Conversely, mortality or another organismal effect without molecular confirmation does not establish sequence-specific RNAi. Protein abundance or activity should be measured when the encoded product is expected to persist or when transcript-level and phenotypic outcomes diverge. Target transcript, encoded protein, and organismal phenotype should therefore be analysed as successive, non-interchangeable endpoints.

A minimum informative design need not incorporate every available analytical method. An initial study should include a route-appropriate measure of exposure, an assessment of extracellular persistence in the relevant compartment, time-resolved quantification of target-transcript abundance by RT-qPCR, and a phenotype mechanistically linked to the target gene. A more mechanistically resolved investigation can also incorporate tissue-specific analyses, tracking of intact dsRNA, cellular or subcellular localisation, sequence-resolved profiling of target-derived small RNAs, and protein-level measurements. Each additional readout should be selected for its capacity to discriminate among the remaining mechanistic explanations rather than included as a generic indicator of methodological sophistication.

### 8.3. Match the Intervention to the Supported Constraint

An intervention should be selected according to the constraint supported by the available evidence, and its evaluation should include a readout of the proposed proximal effect. A stabilising treatment requires demonstration of increased dsRNA persistence in the biological compartment relevant to the selected route. A carrier proposed to improve tissue delivery requires distribution measurements, whereas claims of enhanced cellular entry or endosomal release require cellular or subcellular assays. Greater target-transcript depletion or a stronger phenotype alone cannot identify the altered process because a formulation may simultaneously affect extracellular protection, ingestion, epithelial interactions, tissue distribution, cellular internalisation, and intracellular trafficking [43,44,46,47]. In *N. viridula*, perturbation of a dsRNA-degrading nuclease and the associated change in oral RNAi outcome provided direct evidence that nuclease activity contributed causally under the tested conditions [12]. This process-matched design supports stronger mechanistic inference than improvement in a downstream endpoint alone.

Candidate delivery technologies examined in insects include chitosan-based carriers, liposomes, star polycations, carbon quantum dots, layered double hydroxides, guanylated polymers, branched amphiphilic peptide capsules, and cell-penetrating peptides [46]. Evidence obtained outside Heteroptera constitutes class D evidence: it can support selection of a candidate technology and formulation of a mechanistic hypothesis but cannot demonstrate that the corresponding constraint or carrier effect occurs in heteropteran insects. For example, endosomal accumulation and lipid-facilitated release demonstrated in *S. frugiperda* [41,42] define the measurements required to test this process in Heteroptera; they do not establish endosomal entrapment or carrier-mediated escape in this suborder. Similarly, experiments using a heteropteran species solely as a non-target organism inform biosafety rather than carrier efficacy.

Several formulation systems have now been examined directly in Heteroptera, although they provide varying degrees of mechanistic resolution. In *E. heros*, liposome-based and EDTA-containing formulations increased dsRNA protection and oral mortality [11]. These findings support formulation-dependent preservation of dsRNA and an improved biological outcome but do not isolate protection from possible effects on ingestion, epithelial passage, or subsequent internal exposure. In *H. halys*, dsDNA co-formulation reduced dsRNA degradation in saliva and increased target-transcript depletion [14]. The absence of a corresponding phenotypic improvement indicates that greater extracellular persistence and transcript depletion do not necessarily overcome constraints operating between transcript and organismal phenotype. Neither system establishes a generally effective stabilisation strategy across Heteroptera.

A chitosan-based system evaluated in *A. lucorum* included a particularly informative set of controls: carrier only, naked target dsRNA, non-target dsRNA, and formulated non-target dsRNA [20]. This design strengthens the distinction between sequence-specific RNAi, carrier toxicity, and non-specific formulation effects. Physicochemical characterisation and resistance to RNase A supported complex formation and protection under the tested model conditions, but protection was not assessed in native midgut fluid. Moreover, the fluorescence experiment tracked labelled chitosan rather than intact dsRNA. The molecular and phenotypic responses therefore support productive completion of the delivery and silencing sequence as a whole but do not directly demonstrate improved tissue delivery, cellular internalisation, or endosomal release. A proton-sponge mechanism remains a proposed explanation rather than a directly tested process in this system.

SPc complexation has also been examined in *A. lucorum*. In contrast to the earlier chitosan study, protection was demonstrated in native midgut fluid, providing direct evidence of a proximal effect on extracellular dsRNA persistence [18]. Molecular and phenotypic responses were subsequently observed after direct topical application and spraying onto plants infested with nymphs. However, the downstream improvement cannot be attributed specifically to protection because SPc may affect several formulation- and delivery-related processes. The spraying treatment also combined potentially distinct exposure pathways and did not isolate direct contact from ingestion of dsRNA from treated plant material. Fluorescence or whole-insect estimates of dsRNA-associated material do not, on their own, establish the distribution of intact dsRNA, cellular entry, or cytoplasmic release. Endosomal escape therefore remains unresolved for both chitosan- and SPc-based systems.

Plant-mediated delivery should be treated as a distinct exposure strategy rather than a nanocarrier technology. In *A. lucorum*, Liu et al. [19] demonstrated target-transcript depletion and phenotypic effects in insects fed on transgenic plants expressing target dsRNA. Silencing of the same target after injection provided complementary evidence that productive RNAi could occur under both experimental conditions. However, the biologically available dose and resulting internal exposure were unknown, and the effects obtained through the two routes cannot be compared quantitatively. The plant-mediated result demonstrates functional delivery within that exposure context but does not, by itself, identify improved dsRNA stability, uptake, or intracellular processing.

Intervention studies should therefore follow an explicit perturbation–response sequence: the candidate constraint should first be documented for the relevant route and biological context; an intervention expected to alter that process should then be applied; its proximal effect should be measured directly; and downstream changes in target transcript, encoded protein, and organismal phenotype should be assessed as separate endpoints. If extracellular persistence increases without greater target-transcript depletion, subsequent experiments should examine internal exposure, tissue access, or intracellular processing rather than continue optimising protection alone. If target-transcript depletion increases without the predicted phenotype, further work should address tissue specificity, depletion duration, protein persistence, functional thresholds, and endpoint selection. This sequence prevents a specific mechanism from being assigned to a successful formulation solely on the basis of its final molecular or phenotypic outcome.

### 8.4. Align Target, Tissue, Timing, Endpoint, and Controls

Target-gene selection cannot be separated from route-dependent accessibility and biological interpretation. A suitable target should be expressed in the tissue and developmental window relevant to the intended effect, and its depletion should be expected to alter a measurable biological function [2,43,44,45,46,47]. Injection-induced target-transcript depletion demonstrates that the construct can produce silencing in at least some responsive cells under the tested conditions. When accompanied by the predicted phenotype, this can support the use of the target in functional genomics but does not establish its accessibility or suitability for oral, plant-mediated, or topical control. Conversely, a target accessible through the intended practical route may remain useful even if its depletion does not cause rapid mortality, provided that it reduces feeding, development, reproduction, or another fitness-related trait.

Informative endpoints are necessarily target-specific. Studies of Hox genes in *O. fasciatus*, *nitrophorin 2* in *R. prolixus*, *vitellogenin* in *C. lectularius*, and *white* in *N. viridula* assessed developmental, salivary, reproductive, and pigmentation outcomes rather than using mortality as a universal criterion [21,23,24,35,36,37]. Mortality remains relevant for pest-oriented targets but should be accompanied by molecular confirmation of target-transcript depletion and by non-lethal endpoints when target biology predicts delayed or fitness-related effects [2,43,45,47]. The expected direction and temporal onset of each molecular and biological response should be specified before exposure.

Sampling schedules should reflect anticipated transcript kinetics, protein persistence, developmental transitions, and the threshold required to produce the selected phenotype [2,24,43,45]. Early and late measurements can distinguish transient target-transcript depletion from delayed pathway engagement, subsequent transcript recovery, or a phenotype that emerges only after depletion of the encoded protein. When the relevant function is confined to a discrete organ or tissue, tissue-specific RT-qPCR or another tissue-resolved assay should take precedence over whole-body measurement alone [22,43,44,45]. Substantial local depletion may be obscured by whole-body analysis, whereas an apparent whole-body response may occur predominantly outside the tissue responsible for the phenotype. Protein abundance or activity should also be measured when the encoded product is expected to persist or when transcript depletion does not produce the predicted biological effect [2,43,45].

Controls should align directly with the inference being tested. Experiments require an untreated baseline, an appropriate route-specific vehicle or procedural control, and a non-target dsRNA control. Injection experiments require a control for the injection procedure. Carrier studies should include carrier-only, naked target dsRNA, non-target dsRNA, and formulated non-target dsRNA treatments, together with a dose-matched formulated target-dsRNA treatment [45,46,47]. This design distinguishes sequence-specific silencing from carrier toxicity, altered exposure, and other formulation-associated effects. Feeding and plant-mediated studies should quantify food intake or another validated indicator of feeding whenever treatment-related changes in ingestion could affect either the biologically available dose or the measured phenotype [2,43,47]. Concordant molecular and biological responses obtained with two independent, non-overlapping fragments of the target gene, or another independent sequence-specific validation, further strengthen causal attribution by reducing the likelihood of off-target or construct-specific explanations [43,45,46,47].

The plant-mediated experiment by Liu et al. [19] also highlights an important control limitation. The transgenic maize and soybean lines expressing target dsRNA were compared with wild-type plants. Wild-type plants provide a baseline for insect performance on plants not producing the target dsRNA, but they do not replace a transgenic non-target-dsRNA control and therefore do not isolate sequence-specific RNAi from possible transformation-, transgene-expression-, or plant-line-associated effects. Future HIGS studies should include a matched transformed line or, preferably, a plant producing a sequence-irrelevant dsRNA at a comparable level. Multiple independent target-dsRNA lines remain valuable for evaluating line-to-line consistency, but they do not substitute for this control.

Optimisation should proceed iteratively by testing a defined intervention and measuring both its proposed proximal action and the resulting molecular and organismal outcomes. Conclusions should be limited to the species, developmental stage, delivery route, construct, tissue, sampling period, and endpoint examined; transfer to another experimental context requires renewed diagnostic evaluation.

## 9. Conclusions and Future Research Priorities

The available evidence does not support characterising Heteroptera as uniformly refractory to RNAi. Target-transcript depletion and biologically informative phenotypes following injection, together with selected oral, plant-mediated, topical, systemic, and maternal responses, demonstrate functional RNAi competence in multiple experimental systems [9,13,18,19,20,21,23,24,30,31,32,33,34,35,36,37]. This competence is context-dependent: outcomes vary with delivery route, dose, formulation, developmental stage, tissue, target gene, sampling time, and endpoint. RNAi responsiveness should therefore be defined for a specified experimental context rather than assigned as a fixed property of a species, family, feeding guild, or the suborder as a whole.

Process-specific evidence remains unevenly distributed. The most extensive body of evidence remains concentrated in Pentatomidae and concerns extracellular dsRNA degradation, formulation-dependent protection, and selected systemic or maternal responses [10,11,12,13,14,24]. However, the evidence base for Miridae is broader than previously recognised. It includes salivary and haemolymph-associated degradation in *L. lineolaris*, gut-fluid degradation and recombinant-nuclease activity in *A. lucorum*, and functional RNAi responses after injection, plant-mediated exposure, chitosan-assisted oral delivery, direct topical application, and plant spraying [16,17,18,19,20]. These findings substantially extend the range of processes and delivery contexts examined in Miridae but do not match the overall concentration and mechanistic depth of studies in Pentatomidae. They also remain insufficient to rank families or species by intrinsic RNAi responsiveness.

Direct causal evidence is considerably less abundant than reports of molecular or phenotypic responses. Nuclease perturbation has linked extracellular degradation to an oral RNAi outcome in a defined experimental system, whereas formulation studies have shown that protection can improve downstream responses without necessarily isolating degradation from simultaneous changes in exposure or delivery [11,12,14,18]. Functional access to distant tissues and systemic or maternal effects have also been demonstrated in selected species, but the identity and movement of the biologically active signal remain unresolved [21,23,24]. By contrast, available studies do not establish PMM-mediated restriction, inefficient cellular internalisation, endosomal retention, or deficiencies in individual Dicer- or RISC-associated processes as limiting mechanisms in Heteroptera [9,15,25,26,27,28,29,30]. The multilevel model should consequently be used as a diagnostic framework for testing candidate constraints rather than interpreted as a universal hierarchy of demonstrated barriers.

Future research should prioritise process-specific measurements and matched comparisons across delivery routes using the same construct, developmental stage, principal sampling times, and molecular endpoints. Exposure should be quantified at tissue resolution whenever the biological interpretation depends on access to a particular target site. Sequence-verified, target-derived siRNAs would provide strong evidence of entry into the small-RNA pathway; products identified only by their apparent size remain merely consistent with dsRNA processing [15]. Target-transcript depletion, depletion or activity of the encoded protein, and organismal phenotype should be analysed as distinct, non-interchangeable endpoints. Most importantly, candidate constraints should be tested through causal perturbation accompanied by direct verification of the intervention’s proposed proximal effect.

Progress in both functional genomics and applied pest management will therefore depend on replacing broad classifications of species as RNAi-responsive or RNAi-refractory with evidence-qualified descriptions of defined combinations of species, developmental stage, delivery route, formulation, target gene, target tissue, and endpoint. This approach will distinguish directly demonstrated processes from functional inferences and untested mechanisms, provide a more defensible basis for comparing experimental systems, and clarify which constraints must be overcome before RNAi-based management can be reliably evaluated in heteropteran pests.

## Figures and Tables

**Table 1:** Selected studies of experimentally induced RNA interference in Heteroptera, with molecular and phenotypic endpoints reported separately.

Species (Family)	Delivery/ Intervention	Target(s)	Molecular or Protein Endpoint	Phenotypic/ Functional Endpoint	Evidence- Constrained Interpretation	Reference
*Halyomorpha* *halys* (Pentatomidae)	Micro-injection and feeding	*IAP*, *ATPase*, *SNF7*, *GPCR*, *PP1* and others	Injection reduced selected transcripts by approximately 40–75%; feeding produced target-dependent reductions of approximately 20–60%.	Mortality exceeded 70% for five of 13 injected and three of five feeding constructs.	Both routes produced target-dependent RNAi; the comparison does not identify the limiting step.	[13]
*H. halys* (Pentatomidae)	Micro-injection	*CAT*	Approximately 98% *CAT* transcript reduction.	No significant increase in mortality.	Strong transcript depletion did not translate into mortality, separating RNAi from insecticidal efficacy.	[31]
*H. halys* (Pentatomidae)	Topical application	*IAP*, *ATPase N2B*, *PP1*	Transcript effects varied by target, dose, tissue, and time; some survival effects lacked aligned transcript depletion.	Survival decreased only for selected target–dose combinations.	Topical responses were context-dependent; mortality alone neither confirms target-specific RNAi nor identifies cuticular penetration.	[22]
*H. halys* (Pentatomidae)	Oral dsRNA with dsDNA co-formulant; microinject-ion	*HhCHC*	dsDNA increased ex vivo stability and enabled oral transcript depletion; injection produced stronger depletion.	Oral treatment did not increase mortality; injection produced high corrected mortality.	The co-formulant improved stability and oral knockdown, but degradation was not isolated from other formulation effects.	[14]
*Nezara* *viridula* (Pentatomidae)	Micro-injection and oral feeding	*vATPase A*, *αCop* and other essential genes	Oral *αCop* and *vATPase A* treatments caused target- and time-dependent transcript depletion.	Mortality exceeded 90% for seven of ten injected targets; oral mortality was approximately 45% for *αCop* and 43% for *vATPase A*.	Route differences implicate feeding-associated constraints but do not identify the responsible step.	[10]
*N. viridula* (Pentatomidae)	*NvdsRNase* knockdown followed by oral ds*αCop*	*NvdsRNase*, *αCop*	Strong *NvdsRNase* depletion increased dsRNA stability in saliva and gut fluid ex vivo.	Mortality after oral ds*αCop* increased from approximately 47% to 65%.	Causal evidence that nuclease activity limits oral RNAi; salivary and gut contributions were not separated.	[12]
*Euschistus* *heros* (Pentatomidae)	Micro-injection	*V-ATPase A*	Target depletion accompanied altered *Dcr2* and *Ago2* expression.	Approximately 35% mortality.	Injection supports functional RNAi; component expression does not measure Dicer or RISC efficiency.	[9]
*E. heros* (Pentatomidae)	Oral naked dsRNA and EDTA- or liposome-containing formulations	*vATPase A*, *act-2*	Gut transcript depletion occurred under several treatments but did not consistently track mortality.	Liposomes produced approximately 45% and 42% mortality for *vATPase A* and *act-2*, respectively; EDTA effects were target-dependent.	Selected outcomes improved, but effects on protection, uptake, and intracellular processing were not isolated.	[11]
*Piezodorus* *guildinii* (Pentatomidae)	Micro-injection	*V-ATPase A*	Transcript depletion of approximately 64–84% persisted for 24–72 h.	Mortality reached approximately 52% by day 14.	Injection supported sustained functional knockdown; individual intracellular steps were not measured.	[30]
*Plautia stali* (Pentatomidae)	Micro-injection and oral feeding	*MCO2-2*, *vATPase*, *IAP*, *SNF7*	Injection caused 80–99.9% depletion at the higher dose; oral effects were limited to selected targets at the highest concentration.	Injection caused developmental or survival phenotypes; oral effects were weak or absent.	Route-, dose-, and target-dependent responses do not identify the constraint on oral delivery.	[32]
*Cimex* *lectularius* (Cimicidae)	Micro-injection	*ClVg*	Strong *ClVg* transcript depletion.	Markedly reduced egg production, ovarian atrophy, and fat-body hypertrophy.	Injection revealed reproductive function but did not characterise biodistribution.	[23]
*C. lectularius* (Cimicidae)	Micro-injection and topical application	*Muscle* *actin*	Injection caused transcript depletion; topical application did not.	Injection caused greater than 80% mortality by day 20 and inhibited oviposition; topical treatment had no comparable effect.	The route effect is functional evidence but does not distinguish penetration, stability, distribution, or cellular entry.	[33]
*C. lectularius* (Cimicidae)	Micro-injection	*ClCPR*	Reduced *ClCPR* transcript abundance.	Increased insecticide susceptibility, particularly in resistant populations.	The phenotype depended on population background and insecticide challenge, not only knockdown magnitude.	[34]
*Rhodnius* *prolixus* (Reduviidae)	Ingestion and micro-injection	*NP2*	Salivary-gland *NP2* expression decreased by 42 ± 10% after ingestion and 75 ± 14% after injection.	Treated-insect saliva prolonged coagulation less than control saliva.	Oral dsRNA affected a distant tissue; the mobile signal and route remain unresolved.	[21]
*Oncopeltus* *fasciatus* (Lygaeidae)	Embryonic and/or nymphal micro-injection	*Dfd*, *pb*, *Scr*, and other Hox genes	Target-transcript depletion was not measured.	Reproducible gene-associated phenotypes revealed embryonic and post-embryonic Hox functions.	Phenotypes support gene-associated effects but do not provide molecular confirmation or quantify pathway efficiency.	[35,36,37]
*Lygus* *lineolaris* (Miridae)	Oral long ds*IAP* or *IAP*-derived siRNA in membrane sachets	*IAP*	No apparent transcript reduction by semi-quantitative RT-PCR.	Food uptake was confirmed, but no target-specific survival effect was detected.	Oral non-response coincided with salivary degradation, but causality and dose-matched route effects were untested; injection efficacy was shown separately.	[16]
*Apolygus* *lucorum* (Miridae)	Micro-injection screen; plant-mediated ds*V-ATPase-E* via transgenic maize and soybean	Seven candidate genes in the injection screen; *V-ATPase-E* in the plant-mediated experiments	Injection caused target-dependent depletion; feeding on transgenic plants reduced *V-ATPase-E* transcript abundance.	Plant-mediated exposure increased mortality.	Functional plant-mediated RNAi, but exposure and biodistribution were unmeasured; controls used wild-type rather than non-target-dsRNA transgenic plants.	[19]
*A. lucorum* (Miridae)	Chitosan-complexed dsRNA on bean fragments for oral acquisition	*GRK2*	Maximum *GRK2* transcript depletion was approximately 70%.	Increased mortality, reduced body mass, and delayed development.	Functional carrier-assisted oral RNAi; cellular uptake and endosomal escape were not directly measured.	[20]
*A. lucorum* (Miridae)	Direct topical application or spraying onto infested bean plants; dual-target dsRNA–SPc complex	*AlECR-A*, *AlTre-1*	Both target transcripts were depleted at 24 h relative to formulated non-target dsRNA after both treatments.	Mortality reached 69% after direct application and 83% after spraying by day 3.	Direct application supports SPc-formulated topical RNAi, but the improved step was unresolved; spraying allowed mixed exposure, precluding route comparison.	[18]

Note for Table 1. Representative, not exhaustive. Molecular and phenotypic endpoints are distinct; phenotype alone does not confirm target-specific RNAi or its mechanistic basis. Gene symbols follow the cited studies. Abbreviations: dsDNA, double-stranded DNA; dsRNA, double-stranded RNA; EDTA, ethylenediaminetetraacetic acid; RNAi, RNA interference; RT-PCR, reverse-transcription PCR; siRNA, small interfering RNA; SPc, star polycation.

**Table 2:** Evidence classification and diagnostic readouts for processes proposed to influence RNA interference outcomes in Heteroptera.

Process	Evidence Class	Taxonomic Scope	Evidence	Supported Conclusion	Required Diagnostic Readout
Salivary dsRNA degradation	A1–A2 for degradation; C for *AldsRNase* expression	*Lygus lineolaris* and *Apolygus lucorum* (Miridae); *Nezara viridula*, *Euschistus heros*, and *Halyomorpha halys* (Pentatomidae)	In *L. lineolaris*, dsRNA—but not the tested dsDNA—was degraded in saliva, salivary-gland extract, and post-feeding sachet material [16]. Direct degradation was also observed in *N. viridula* and *E. heros*, with causal nuclease evidence in *N. viridula*; results for *H. halys* varied among protocols [10,11,12,13,14]. Salivary-gland expression of *AldsRNase* in *A. lucorum* is candidate-level evidence only [17].	Salivary degradation is directly supported in selected mirids and pentatomids, but its in vivo contribution is system-dependent.	Stage-matched kinetics; intact-dsRNA recovery during feeding; candidate-dsRNase perturbation; matched naked and protected dsRNA.
Gut-fluid dsRNA degradation	A1–A2 in *N. viridula*; A2 in *A. lucorum*	Direct evidence: *N. viridula* (Pentatomidae) and *A. lucorum* (Miridae); inconclusive candidate-nuclease test: *Plautia stali* (Pentatomidae)	Native midgut-fluid degradation was shown in *N. viridula* and *A. lucorum*. *NvdsRNase* depletion increased stability only in *N. viridula* [10,12]. Purified AldsRNase degraded dsRNA without causal perturbation [17]. Qiao et al. [20] showed chitosan protection against RNase A and physicochemical stress, not native fluid; Qiao et al. [18] showed native-fluid degradation and protection. The *P. stali* co-feeding test was inconclusive [32].	Gut-fluid degradation is directly supported in *N. viridula* and *A. lucorum*. Nuclease causality is established only in *N. viridula*; multifunctional-carrier protection does not identify the process improving RNAi.	Stage-matched native-fluid kinetics; intact-dsRNA recovery in vivo; separate salivary and gut-fluid assays; dsRNase perturbation with matched RNAi endpoints.
Haemolymph- associated degradation	A2: direct mechanistic observational evidence	*L. lineolaris* (Miridae); *E. heros* and *H. halys* (Pentatomidae)	Ex vivo haemolymph degradation was reported in *E. heros* and *H. halys*, with assay-dependent rates [9,13,14,15]. In *L. lineolaris*, dsRNA remained detectable after 1 h but was degraded after overnight incubation, indicating slower ex vivo activity than that observed in saliva under the assay conditions [16].	Haemolymph degradation occurs in selected species; its in vivo rate and effect on systemic persistence remain unresolved.	In vivo half-life; compartment-specific intact-dsRNA recovery; matched tissue-exposure and silencing time courses.
Midgut interface, including the perimicrovillar membrane (PMM)	E: anatomical context; transport untested	Multiple heteropteran families; anatomical evidence only	The PMM and heteropteran midgut architecture are anatomically documented, but dsRNA permeability, retention, and transport were not measured [25,26,27,28,29].	The PMM is a plausible interface, not a demonstrated barrier to dsRNA movement.	Tracking of intact dsRNA across the PMM/epithelium; permeability or recovery assays; interface perturbation linked to tissue-resolved knockdown.
Cuticular passage and topical internal exposure	B–C: functional and indirect route-level evidence; mechanism unresolved	*A. lucorum* (Miridae); route-level outcomes in *H. halys* and *Cimex lectularius*	In *A. lucorum*, SPc increased dsRNA-associated fluorescence and estimated whole-insect dsGFP after topical application; formulated target dsRNA caused knockdown and mortality [18]. Topical outcomes varied in *H. halys* and *C. lectularius* [22,33].	Carrier-assisted topical RNAi can succeed, but the amount and integrity of dsRNA crossing the cuticle and the role of cuticular passage remain unresolved.	Removal of surface-bound dsRNA; integrity-sensitive internal recovery; depth-resolved imaging; matched naked/formulated dsRNA; cuticular perturbation linked to knockdown.
Tissue distribution and systemic movement	B–C: functional systemic effects; indirect carrier tracking	Reduviidae, Pentatomidae, Cimicidae, and Miridae; few species and tissues	Oral or injected dsRNA produced non-local, multi-tissue, maternal, or reproductive effects in *Rhodnius prolixus*, *N. viridula*, and *C. lectularius* [21,22,23,24]. Qiao et al. [20] tracked labelled-chitosan fluorescence in *A. lucorum*, not dsRNA.	Non-local effects can occur, but the mobile signal and its distribution remain unresolved; carrier fluorescence cannot establish dsRNA movement.	Organ-level tracking of intact dsRNA and target-derived siRNAs; tissue-specific knockdown; haemolymph sampling; local-versus-systemic transfer assays.
Cellular uptake	B–C: functional or indirect evidence; uptake unmeasured	Pentatomidae and Miridae; functional observations in other heteropterans	Candidate endocytic components occur in *E. heros* and *Piezodorus guildinii* [9,30]. In *A. lucorum*, knockdown after chitosan-assisted feeding [20] and SPc-assisted topical/mixed exposure [18] supports productive completion, but carrier fluorescence and transcriptional responses did not measure dsRNA entry. Other knockdown and siRNA-sized-product observations provide indirect support [14,15].	Productive entry is functionally supported, including in carrier-assisted *A. lucorum* systems, but its route and efficiency are unmeasured and limited uptake is not a demonstrated barrier.	Quantitative uptake in cells or ex vivo tissues; cellular intact-dsRNA tracking; pathway co-localisation and perturbation linked to cytoplasmic delivery and knockdown.
Endosomal trafficking and cytoplasmic release	E: unresolved in Heteroptera	No direct evidence; proposed carrier mechanisms in *A. lucorum* (Miridae)	The proton-sponge effect is a proposed mechanism for cationic-carrier-mediated release. Neither chitosan [20] nor SPc [18] was tested directly for endosomal localisation, trafficking, or cytoplasmic release in *A. lucorum*.	Endosomal escape is plausible but untested; carrier-associated responses do not demonstrate cytoplasmic release.	Endosomal/lysosomal co-localisation; subcellular fractionation; cytoplasmic intact-dsRNA or target-siRNA quantification; escape-specific perturbation with stability/uptake controls.
Intracellular processing and core siRNA machinery	B–C/E: downstream function and candidates supported; individual steps untested	Pentatomidae and Miridae; downstream observations in other heteropterans	Core-pathway transcripts and unverified siRNA-sized products provide candidate-level evidence [9,15,30]. In *A. lucorum*, knockdown after plant-mediated feeding [19], chitosan-assisted feeding [20], and SPc-assisted topical/mixed exposure [18] is downstream functional evidence only. Dicer-2 processing, target-derived siRNAs, AGO2 loading, and RISC cleavage were not measured.	Core candidates and downstream silencing are supported, but individual steps and efficiencies remain unresolved; no general pathway-level defect is demonstrated.	Target-specific small-RNA sequencing; Dicer-2 assays; AGO2 immunoprecipitation/guide loading; tissue-specific siRNA and knockdown time courses.
Transcript-to-protein and phenotype translation	B: direct functional evidence	Pentatomidae, Miridae, Cimicidae, and Reduviidae; several species and targets	Transcript depletion and phenotype were sometimes discordant [13,14,22,23,24,31,32,33,34]. In *A. lucorum*, both endpoints were concordant after plant-mediated and chitosan-assisted feeding [19,20] and SPc-assisted topical/mixed exposure [18], although the plant-mediated study had limited controls.	Concordance supports functional completion, not confirmation of Dicer-2, RISC, or another pathway step. Sequence-specific attribution requires adequate controls.	Matched transcript, protein, and phenotype time courses; target-appropriate endpoints; non-target dsRNA, formulation, and toxicity controls.

Note for Table 2. Evidence classes refer exclusively to evidence obtained in Heteroptera: A1, direct mechanistic evidence with causal perturbation; A2, direct mechanistic observational evidence; B, direct functional evidence that does not identify the responsible step; C, indirect or candidate-level evidence; and E, unresolved owing to the absence of a direct test. Evidence from other insect orders may inform diagnostic hypotheses but does not increase the assigned evidence class. Composite labels indicate that different subclaims within a process are supported at different evidence levels; they do not constitute additional intermediate evidence classes. Abbreviations: dsRNA, double-stranded RNA; PMM, perimicrovillar membrane; RISC, RNA-induced silencing complex; RNAi, RNA interference; siRNA, small interfering RNA.

## Data Availability

No new data were created or analyzed in this study. Data sharing is not applicable to this article.

## Abbreviations

The following abbreviations are used in this manuscript:

AGO2	Argonaute 2
Cy3	cyanine 3
dsDNA	double-stranded DNA
dsRNA	double-stranded RNA
EDTA	ethylenediaminetetraacetic acid
HIGS	host-induced gene silencing
PMM	perimicrovillar membrane
RdRP	RNA-dependent RNA polymerase
RISC	RNA-induced silencing complex
RNAi	RNA interference
RNase A	ribonuclease A
RT-qPCR	reverse-transcription quantitative PCR
SID	systemic RNA interference deficient
SIGS	spray-induced gene silencing
siRNA	small interfering RNA
SPc	star polycation
